# Combining Niche Breadth to Predict the Current and Future Distribution of Leguminosae Under Climate Change on the Qinghai‐Xizang Plateau

**DOI:** 10.1002/ece3.71895

**Published:** 2025-08-21

**Authors:** Sen‐Xin Chai, Hui‐Yuan Ma, Chen‐Di Wang, Yan‐Gang Ying, Dong Han, Yue Zhong, Bo Wang, Yuan‐Ming Xiao, Ying Yang, Guo‐Ying Zhou

**Affiliations:** ^1^ CAS Key Laboratory of Tibetan Medicine Research Northwest Institute of Plateau Biology Xining China; ^2^ College of Life Sciences Qinghai Normal University Xining China; ^3^ Qinghai Traffic Construction Management Co., Ltd. Xining China; ^4^ University of Chinese Academy of Sciences Beijing China; ^5^ Qinghai University Xining China

**Keywords:** ecological niche, eight Leguminosae species, MaxEnt model, potential distribution

## Abstract

The Leguminosae family plays a significant role in life and serves as an important food crop. However, global warming poses a serious threat to the growth and potential distribution of Leguminosae species on the Qinghai‐Xizang Plateau. In this study, we employed the MaxEnt model alongside ecological niche models (ENMtools) to predict the distribution of Leguminosae in the Qinghai‐Xizang Plateau under various climate scenarios (RCP2.6, RCP6.0, RCP8.5) for both the present (Current) and future (2050s, 2070s). This analysis was conducted in conjunction with ecological niche principles. The results showed that: (1) The AUC values of the eight Leguminosae species were all greater than 0.9, indicating that the model had a good prediction accuracy. (2) The distribution of the eight Leguminosae species was primarily influenced by altitude. *S. alopecuroides* and *C. korshinskii* were found to be suitable for growth at lower altitudes (Approximate range 1600 ~ 2000 m). In contrast, *A. mongholicus* , *G. uralensis* , and *M. ruthenica* were suitable for growth at high altitudes (Approximate range 2300 ~ 2550 m). (3) The ecological niche of *S. alopecuroides* was found to be largest (B1 = 0.16, B2 = 0.92), whereas that of *G. uralensis* was smallest (B1 = 0.06, B2 = 0.87). Meanwhile, *M. ruthenica* exhibited the highest ecological niche overlap with *M. sativa* (*D* = 0.69, *I* = 0.92), followed by *M. sativa* and *M. officinalis* (*D* = 0.65, *I* = 0.88), and the lowest overlap was observed between *M. sativa* and *S. salsula* (*D* = 0.40, *I* = 0.67). Regarding range overlap, *G. uralensis* demonstrated a significant degree of overlap with most species, particularly with *S. salsula*, which had the highest overlap value (0.81). Conversely, *S. salsula* exhibited relatively low range overlap with most species, with *G. uralensis* and *M. officinalis* had the lowest range overlap (0.25). (4) Under future global warming climate scenarios, the suitable habitat for *M. ruthenica* is projected to decrease, while the suitable habitat for the other seven Leguminosae species is expected to increase to varying extents. This study can provide a reference for species conservation of Leguminosae in the Qinghai‐Xizang Plateau and the planning of species conservation areas.

## Abstract 2

The Leguminosae family plays a significant role in our lives and serves as an important food crop.

## 1 Introduction

According to the Sixth Assessment Report of the Intergovernmental Panel on Climate Change (IPCC) (Butler et al. 2016), the global average temperature has warmed by 0.74°C, and will increase by approximately 2°C–4°C by the end of 2100 (He et al. 2019). Climate change can significantly influence the distribution of surface vegetation, thereby impacting overall landscape patterns (Fernandez et al. 2015). Alpine plants are more sensitive and vulnerable to climate change. Consequently, it is essential to accurately predict the distribution of suitable habitats for protected plant species across spatial and temporal scales under future scenarios (Remya et al. 2015). The Qinghai‐Xizang Plateau, often referred to as the “Third Pole of the Eart,” significantly influences the distribution patterns of numerous species due to its unique habitat. Most plants in this region are extremely sensitive to changes in the environment because of their specialized habitats (Sony et al. 2018). Alpine plants are adapted to low‐temperature environments (Yw et al. 2020), and their populations may decline or even become extinct due to rising temperatures. Researchers have demonstrated that rising global temperatures will impact the survival and geographic distribution of plant species on the Qinghai‐Xizang Plateau, necessitating their migration to higher altitudes or latitudes (Chen et al. 2011). The distribution of alpine plant species is highly sensitive to environmental factors (Li et al. 2018). For example, *Meconopsis* Vig is an alpine plant whose survival is determined by altitude; thus, it cannot be grown in plains or low‐altitude areas (Wang et al. 2021). Similarly, the most significant environmental factor affecting the *Pomatosace filicula* Maxim is elevation, as this species can only migrate to higher, more humid locations in the future (Chen et al. 2022).

Ecological niches measure the ability of plants to adapt to their environment and are one of the determinations of how well a plant has adapted to its environment (Yan et al. 2024). However, the rate of niche adjustment in alpine plants may be slower than the rate of climate change (Chen, Lu, et al. 2024; Chen, Wang, et al. 2024), and species with broader niches tend to have more extensive habitats. For instance, *Rheum moorcroftianum* Royle can adapt to poorer environmental conditions owing to its wider ecological niche, resulting in a larger growth range (Chen, Lu, et al. 2024; Chen, Wang, et al. 2024). Furthermore, overlapping ecological niches among species can lead to habitat competition; species with limited resources that share the same ecological niches and resource utilization patterns are unlikely to coexist in the long term (Xiao et al. 2024). Thus, understanding the ecological niches of species is vital for prioritizing conservation efforts and elucidating the phenomena of habitat increases or decreases among species (Peterson et al. 2011; Sexton et al. 2017).

There are approximately 650 genera of Leguminosae worldwide, with 172 genera found in China, distributed across all provinces and regions (Veitch 2007). Leguminosae are economically significant, serving as a vital source of starch, protein, oil, and vegetables for human consumption (Hughes 2017). Leguminosae growing on the Qinghai‐Xizang Plateau can also be used in the field of vegetation restoration. Jiang et al. (2023) investigated *Caragana korshinskii* Kom, with their findings offering insights into the potential applications of this species for vegetation restoration in desert areas. Research has demonstrated that Leguminosae plants can form symbiotic relationships with soil rhizobacteria during their growth, enabling them to utilize environmental nitrogen (Franche et al. 2009; Kazydub et al. 2020; Atlantis Press 2019). Furthermore, the biomass of Leguminosae species serves as a direct indicator of ecosystem stability, with several species being widely employed in ecological restoration efforts (Bargali 2016; Li et al. 2020; Jiang et al. 2020). While the geographical distribution of Leguminosae species has been extensively studied at national and global levels, understanding their distribution on the Qinghai‐Xizang Plateau is essential for species conservation and identifying priority areas for protection (Wei et al. 2021; Ma and Sun 2018).

**FIGURE 1:**
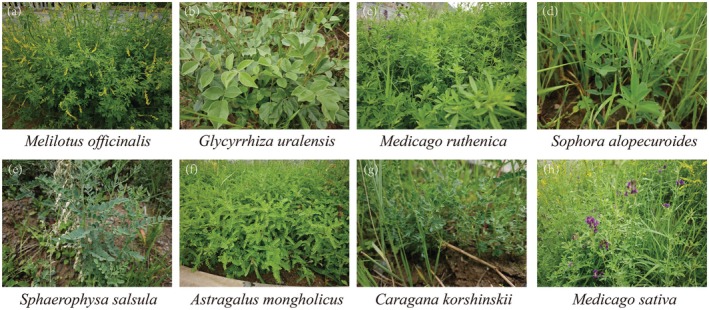
Plant morphology photo of the eight Leguminosae species.

In this study, we selected eight Leguminosae species that are prevalent on the Qinghai‐Xizang Plateau (Figure 1). These species include *M. sativa* L recognized as the “king of pasture” on the plateau; *A. mongholicus*, a common medicinal plant known for replenishing vital energy; *G*. *uralensis*, referred to as the king of medicines; and *M*. *officinalis*, an excellent pasture grass that contributes to soil and water conservation (Shi et al. 2017; Ren et al. 2023; Wang et al. 2018; Al‐Snafi 2020). Predicting the current and future distribution of these Leguminosae on the Qinghai‐Xizang Plateau under climate change scenarios using geographic distribution and environmental data (Fitzpatrick et al. 2013; Qin and Li 2023). The objectives of this study were to: (1) assess the current distribution of Leguminosae in the context of global warming; (2) analyze the primary environmental factors influencing the distribution of Leguminosae; (3) elucidate the changes in the extent of suitable habitat under future climate change; and (4) clarify the ecological niche breadth for each species, as well as the degree of ecological niche and geographic range overlap among the species, understanding the relationship between their ecological niche and changes in suitable habitat. This study can provide scientific insights for in situ conservation and rational utilization of Leguminosae growing on the Qinghai‐Xizang Plateau.

## 2 Materials and Methods

### 2.1 Data and Variable Sources

**FIGURE 2:**
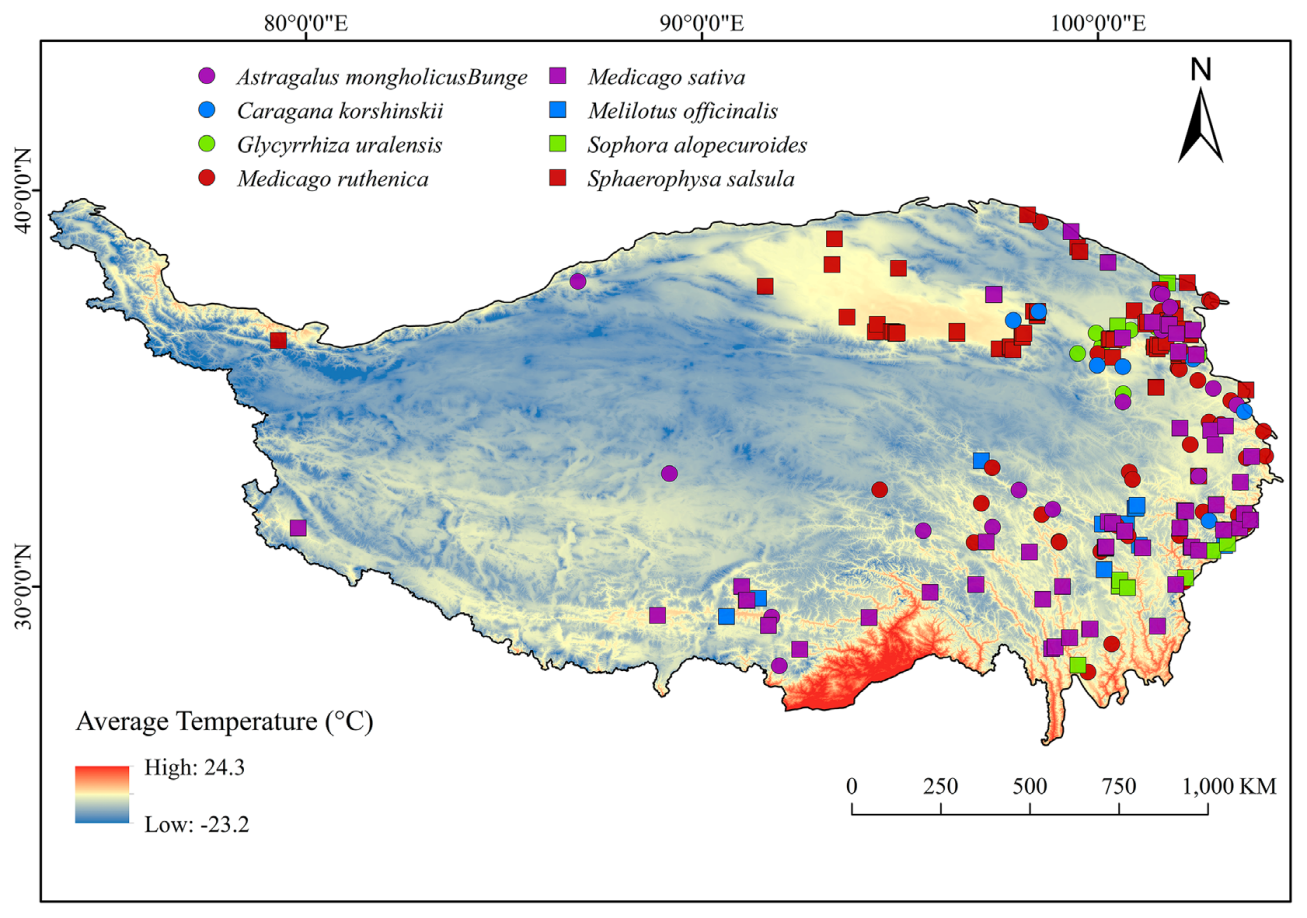
Modern occurrence sites of the eight Leguminosae species.

In order to obtain the distribution records of eight Leguminosae on the Oinghai‐Xizang Plateau, this study consulted the CNKI database (http://www.cnki.net/ accessed on 7 October 2022); China Digital Herbarium (https://www.cvh.ac.cn/ accessed on 7 October 2022); NSII China National Herbarium Resource Leveling (http://www.nsii.org.cn/2017/home.php accessed on 7 October 2022); Global Biodiversity Information Facility (https://www.gbif.org/zh/); China Plant Image Library (http://ppbc.iplant.cn/ accessed on 7 October 2022) (Syfert et al. 2013; Anderson and Gonzalez 2011; Gong et al. 2022). In the absence of precise latitude and longitude coordinates, we were the latitude and longitude coordinates by inputting the geographic location of descriptions into Google Earth (http://ditu.google.cn/ accessed on 7 October 2022) and in order to avoid the effects of spatial autocorrelation due to the proximity of sample points, the redundant points were removed using ENMTools. Spatial autocorrelation effects, redundant points were removed using ENMTools (Table 1), and the latitude and longitude coordinates of the samples were stored in an Excel sheet and converted into CSV format for constructing the MaxEnt model (Qin and Li 2023) (Figure 2).

**TABLE 1:** Geographical range and plot information of eight species of Leguminosae on the Oinghai‐Xizang Plateau.

Species	Plot number	Optimized point number	Longitudinal range (°E)	Latitudinal range (°N)
*Sophora alopecuroides*	37	34	75.4–104.7	25.0–38.9
*Medicago ruthenica*	100	80	94.5–104.6	25.0–39.0
*Glycyrrhiza uralensis*	48	43	94.9–105.1	27.3–38.6
*Melilotus officinalis*	27	27	90.6–105.8	28.8–36.9
*Sphaerophysa salsula*	162	104	76.0–104.8	29.3–40.0
*Astragalus mongholicus*	43	41	83.0–104.6	28.0–37.4
*Caragana korshinskii*	25	22	97.9–103.8	25.0–39.4
*Medicago sativa*	128	91	79.8–103.9	24.3–38.9

In this study, we used 28 environmental variables, of which we downloaded 19 bioclimatic factors and elevation variables from the WorldClim Global Climate Database (version 1.4) (http://www.worldclim.org/ accessed on 7 October 2022) and 8 soil variables at the National Tibetan Plateau Data Center (https://data.tpdc.ac.cn/home accessed on 7 October 2022), and it has a spatial resolution of 30 arcseconds (about 1 km). In order to reduce the download volume of raster data in WorldClim 1.4 (htps://www.worldclim.org/data/v1.4/fomrmats.html), the temperature‐related data are processed by multiplying the normal temperature value by 10. Therefore, in this paper, we analyzed the temperature‐related data, the annual mean temperature (bio1), the daily difference of mean temperature (bio2), the isothermality (bio3), the seasonal coefficient of temperature change (bio4), maximum temperature of the warmest month (bio5), minimum temperature of the coldest month (bio6), annual difference of temperature (bio7), mean temperature of the wettest season (bio8), mean temperature of the driest season (bio9), mean temperature of the warmest season (bio10), and mean temperature of the coldest season (bio11) (Li et al. 2020). The raster data values were divided by 10 using the AreGIS 10.8 “Raster Analysis‐Divide” tool, allowing them to be analyzed simultaneously with the data in Wonldclim2.1. The soil variables were resampled and processed. In order to eliminate the multiple linear relationships between bioclimatic variables, we used EMtools to calculate the Pearson correlation coefficients between 28 environmental variables used in eight Leguminosae, determined 0.8 as the threshold, used the environmental variables with correlation coefficients less than 0.8 to construct the maximum entropy model, and utilized the maximum entropy model to analyze the contribution of the environmental variables with correlation coefficients. The environmental factors and set the correlation coefficients at a threshold of 0.8, retaining those with correlation coefficients less than 0.8, of which those with correlation coefficients greater than 0.8 were retained with the highest contribution, while removing those with a contribution of zero (Fourcade et al. 2014). The future climate data chose the CCSM4 model, which is currently the most commonly used model, to simulate the response of increasing GHG concentrations to the global climate, and we chose three emission pathways (RCP2.6, RCP6.0, and RCP8.5) with different concentrations to predict the degree of change in the future climate, and all of the bioclimatic climate variables had to be converted to ASCII format for MaxEnt analysis (Table S1).

### 2.2 MaxEnt Parameter Optimization

The model was configured with the following settings: 25% of the samples were designated as the test set and the remaining 75% as the training set. The number of iterations was specified as 10, and the repeat run type was set to Bootstrap. a “thousand‐point method” was used to assess the impact of environmental factors. The maximum number of iterations was limited to 5000. The output format was selected as “Logistic” and the file type was specified as “ASC.” All other parameters were left at their default values (Moreno‐Amat et al. 2015).

### 2.3 MaxEnt Model Evaluation

To assess the stability of the MaxEnt model assessment, we used a subject operating curve (ROC) to analyze the relative importance of the assessment variables using the Jackknife test; the subject operating curve (AUC) was used to assess the accuracy of the species distribution model, and the area below the ROC curve indicates the magnitude of the AUC, which takes values in the range 0–1. An AUC value below 0.7 indicates poor prediction results, while 0.7 to 0.8 suggests an average outcome. The model is considered better with an AUC value between 0.8–0.9 and excellent with a value exceeding 0.9 (Wei et al. 2019).

### 2.4 Calculation Niche Breadth and Niche Overlap

We used ENMTools v1.4 software to calculate the ecological niche width of each species and the average of Levins B1 (concentration inverse) and B2 (uncertainty) values in the habitat suitability maps of each species. The values of Levins B1 and B2 are in the range of 0–1, and the larger the value indicates that the ecological niche width of its species is larger; vice versa, the species has a narrow ecological niche. We used Schoener's *D* and Hellinger's I indicators to evaluate the degree of ecological niche overlap. The ecological niche overlap was categorized into two components: range overlap among species with the following formula:

$$O_{\mathrm{ij}}=1-0.5\sum_{1}^{n}\left|P_{\mathrm{ia}}-P_{\mathrm{ja}}\right|$$

where Oij denotes the degree of ecological niche overlap between species i and species j, Pia and Pja denote the amount of resources utilized by species i and species j, and Schoener's D and Hellinger's I have values ranging from 0 to 1, with values closer to 1 denoting greater ecological niche overlap between the species, and vice versa with less overlap (Duan et al. 2014).

### 2.5 Classification of Habitat Suitability Ranks

MaxEnt output results were imported into ArcGIS software, and a total of 11 threshold statistics were obtained based on the results of 10 repetitions, and the “10 percentile training presence Logistic threshold” was used as the threshold value to calculate the potential species distribution (Fernandez et al. 2015). Habitat suitability index (0–1), with 0 indicating completely uninhabitable, was calculated by reclassifying eight Leguminosae in ArcGIS software (Table S2).

## 3 Results

### 3.1 Model Selection and Evaluation

**FIGURE 3:**
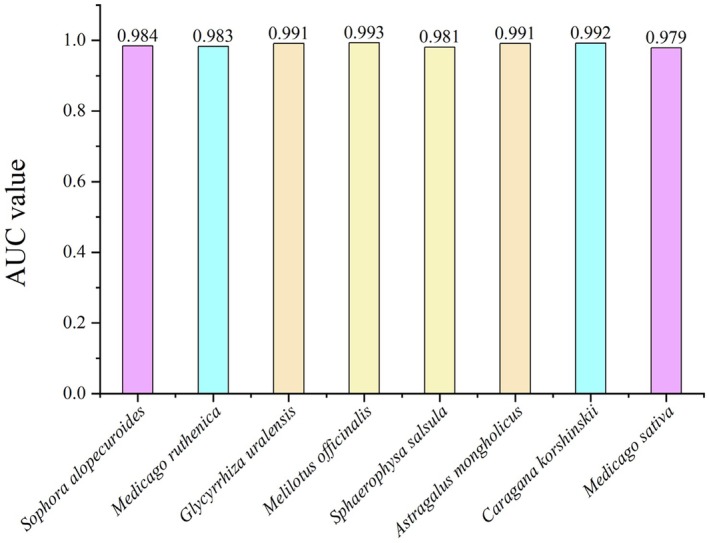
Average test AUC for accuracy analysis of Leguminosae by MaxEnt model under the current situation.

Based on the ROC curve analysis, the average AUC values of current and future predictions of eight Leguminosae species were determined by the average values obtained from 10 replications, and the results showed that: the prediction accuracies of *S. alopecuroides* (AUC = 0.984), *M. ruthenica* (AUC = 0.983), *G. uralensis* (AUC = 0.991), *M. officinalis* (AUC = 0.993), *S. salsula* (AUC = 0.981), *A. mongholicus* (AUC = 0.991), *C. korshinskii* (AUC = 0.992), and *M. sativa* (AUC = 0.979) were better, and could be used for the subsequent analyses. (Figure 3).

### 3.2 Current Potential Distribution of Leguminosae

**FIGURE 4:**
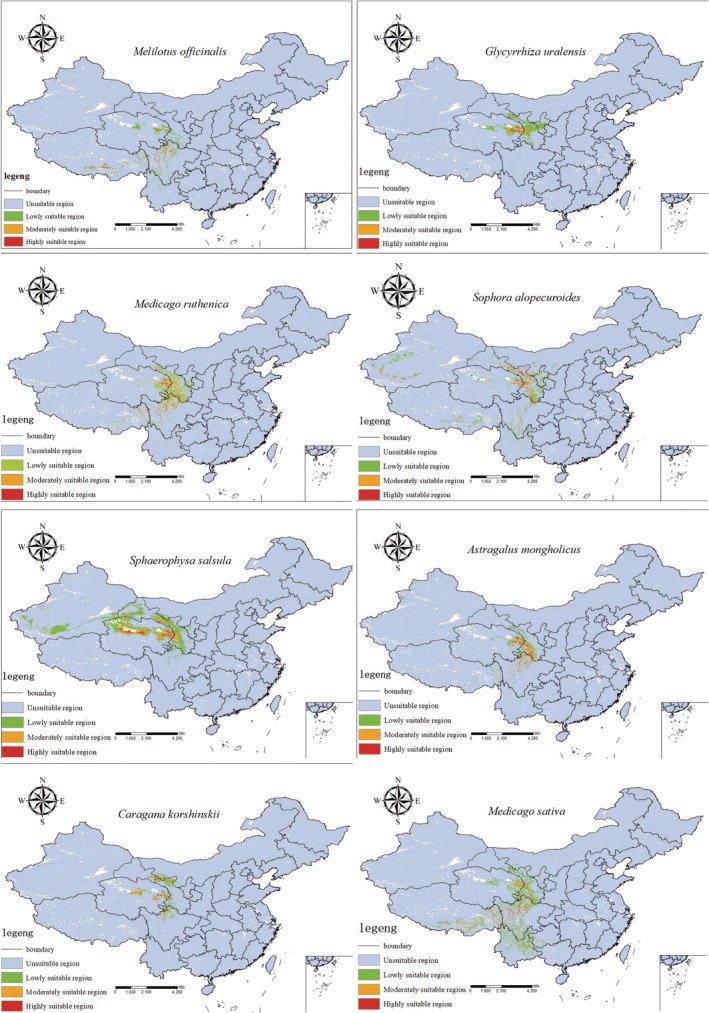
Current distribution of MaxEnt models for eigth species of Leguminosae in China, The color scale from blue to red indicates the habitat suitability value from 0 to 1.

The total area of the habitat of *S. alopecuroides* is 25.26 × 10^4^ km^2^, with the highly suitable area covering 1.01 × 10^4^ km^2^. This habitat is mainly distributed in the eastern part of the Oinghai‐Xizang Plateau, including the Qilian Mountain area, Haidong City, Huangnan Tibetan Autonomous Prefecture, and Gannan Tibetan Autonomous Prefecture. The area of suitable habitat for *M. ruthenica* is 24.73 × 10^4^ km^2^, the area of highly suitable areas is 1.10 × 10^4^ km^2^. This habitat is mainly distributed in the Qilian Mountains, Haidong City, Huangnan Tibetan Autonomous Prefecture, Gannan Tibetan Autonomous Prefecture, Ganzi Tibetan Autonomous Prefecture, and Aba Tibetan Autonomous Prefecture. The area of suitable habitats for *G. uralensis* is 15.48 × 10^4^ km^2^, with the area of highly suitable area being 0.50 × 10^4^ km^2^. The suitable area for *G. uralensis* on the Oinghai‐Xizang Plateau is very small, and the habitat is mainly distributed in Hainan Tibetan Autonomous Prefecture, Huangnan Tibetan Autonomous Prefecture, and Haidong District. The area of suitable habitat for *M. officinalis* is 14.90 × 10^4^ km^2^, with the highly suitable area being 0.96 × 10^4^ km^2^. The habitat is widely distributed, but the suitable habitat is relatively small, with a small portion distributed in Hainan Tibetan Autonomous Prefecture, Huangnan Tibetan Autonomous Prefecture, with a small portion distributed in Ganzi Tibetan Autonomous Prefecture, Gannan Tibetan Autonomous Prefecture, and some areas in southern Tibet. The area of suitable habitat for *S. salsula* is 14.29 × 10^4^ km^2^, with the highly suitable area of 1.89 × 10^4^ km^2^. The habitat is widely distributed, and the area of suitable habitat is large, mainly in Hainan Tibetan Autonomous Prefecture, Huangnan Tibetan Autonomous Prefecture, Haidong District, and Haixi Mongolian Tibetan Autonomous Prefecture, and large areas in Qinghai Province. The area of suitable habitat of *A. mongholicus* is 15.24 × 10^4^ km^2^, with the highly suitable area of 1.19 × 10^4^ km^2^. The distribution of this habitat is concentrated, and the area of highly suitable area is large, mainly distributed in Hainan Tibetan Autonomous Prefecture, Huangnan Tibetan Autonomous Prefecture, Haidong District, and part of Gannan Tibetan Autonomous Prefecture and Aba Tibetan Autonomous Prefecture. The area of suitable habitat of *C. korshinskii* is 13.06 × 10^4^ km^2^, with the highly suitable area of 0.49 × 10^4^ km^2^. The area of this suitable habitat is the smallest among the eight Leguminosae; it is mainly distributed in part of the Qilian Mountains, Huangnan Tibetan Autonomous Prefecture, part of Haidong City, and Haixi Mongol and Tibetan Autonomous Prefecture. The area of the suitable habitat of *M. sativa* is 39.36 × 10^4^ km^2^, with the highly suitable area of 1.24 × 10^4^ km^2^m, the suitable habitat is widely distributed, but the area of the highly suitable zone is relatively small, and the highly suitable zone is mainly located in Huangnan Tibetan Autonomous Prefecture, Gannan Tibetan Autonomous Prefecture, part of Haidong City, and the northwestern part of Sichuan Province (Table 2, Figure 4).

**TABLE 2:** Current habitat composition of eight species of Leguminosae in China under MaxEnt model. HSR, highly suitable region; MSR, medium suitable region; PSR, poorly suitable region; USR, unsuitable region.

Species	Area (*10^4^km^2^)				Percentage (%)			
	HSR	MSR	PSR	USR	HSR	MSR	PSR	USR
*S. alopecuroides*	1.01	6.42	17.83	938.59	0.10	0.67	1.85	97.38
*M. ruthenica*	1.10	5.78	17.85	939.11	0.12	0.60	1.85	97.43
*G. uralensis*	0.50	2.32	12.66	948.37	0.06	0.24	1.31	98.39
*M. officinalis*	0.96	2.67	11.24	948.97	0.10	0.28	1.16	98.46
*S. salsula*	1.89	9.03	3.37	919.56	0.20	0.94	3.46	95.40
*A. mongholicus*	1.19	5.28	8.77	948.60	0.12	0.55	0.91	98.42
*C. korshinskii*	0.49	4.18	8.39	950.79	0.05	0.43	0.87	98.65
*M. sativa*	1.24	7.75	30.37	924.49	0.13	0.80	3.15	95.92

The results showed that most of the suitable areas for the eight Leguminosae species were located in the eastern part of the Qinghai‐Xizang Plateau. Among which, the suitable habitats of *S. salsula* and *M. sativa* were the largest and widely distributed on the Qinghai‐Xizang Plateau, while those of *S. alopecuroides* and *M. officinalis* were more widely distributed, and *C. korshinskii* was the smallest and narrowly distributed. In addition, *M. sativa* overlapped with the rest of the species more often, and *S. salsula* overlapped with the rest of the species less often.

### 3.3 Critical Environmental Factors

**FIGURE 5:**
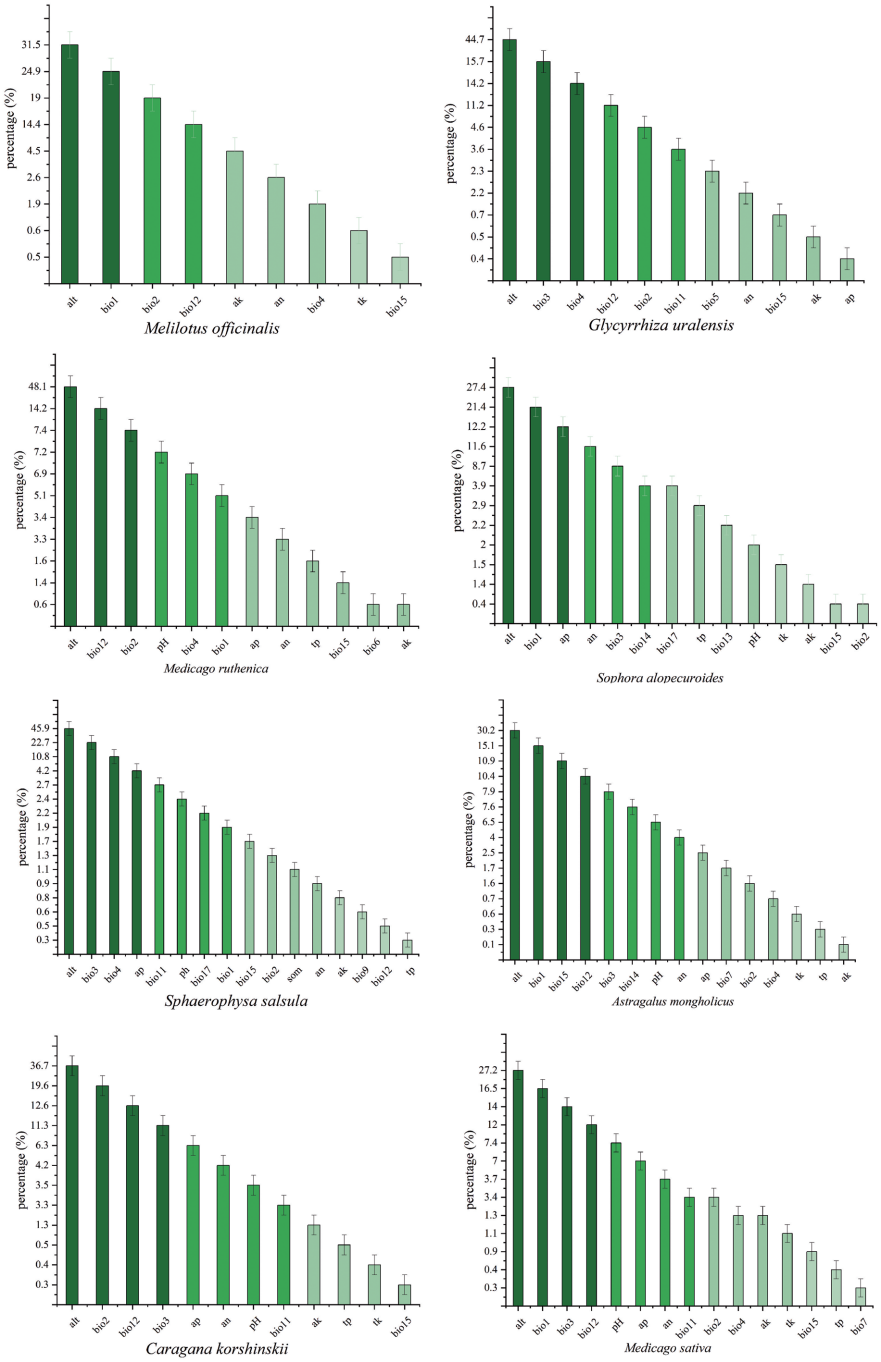
Contribution of environmental factors to the growth and development of eight species of Leguminosae.

We analyzed six contributing factors: altitude (alt), annual mean temperature (bio1), mean monthly temperature (bio2), isothermality (bio3), temperature seasonality (bio4), and annual precipitation (bio12). Among the 28 environmental factors examined, altitude (alt) emerged as the most significant determinant of the distribution of the eight Leguminosae species; the cumulative contribution rate was 36.5%. *S. salsula*, *M. sativa*, and *M. officinalis* exhibited optimal growth at approximately 3000 m above sea level, while *S. alopecuroides* and *C. korshinskii* thrived at lower altitudes (1600 m for *S. alopecuroides* and 2000 m for *C. korshinskii*). *A. mongholicus*, *G. uralensis*, and *M. ruthenica* were found to be suitable for growth at altitudes ranging from 2300 to 2550 m. In addition to altitude, the mean annual temperature (bio1) was identified as the second most important environmental factor influencing the distribution of the four species; the cumulative contribution rate was 19.5%. *A. mongholicus*, *M. sativa*, *M. officinalis*, and *S. alopecuroides*, the likelihood of presence at mean annual temperatures between 6°C and 10°C. Isothermality (bio3) was identified as the third most important environmental factor influencing the distribution of the four species; the cumulative contribution rate was 15.9%. *M. sativa* was the highest probability of presence occurring when isothermality reached 47%. *S. salsula, C. korshinskii*, and *G. uralensis*, with the highest probabilities of species presence observed at isothermality values of 34.5%, 35%, and 36%, respectively. Furthermore, the probability of occurrence for *A. mongholicus*, *M. sativa*, and *M. officinalis* peaked at annual precipitation levels of 600 mm and 700–750 mm. The species exhibited optimal suitability when the coefficient of seasonal variation of temperature reached 9.5 for *S. salsula* and 8.7 for *G. uralensis*, with an optimal mean monthly temperature of 16.2°C for *M. officinalis*. *C. korshinskii* thrives in areas with an average monthly temperature of 15°C–16°C and an annual precipitation of 100 mm. In contrast, *M. ruthenica* is best suited for regions with an average monthly temperature of 15.6°C and an annual precipitation range of 600–800 mm. Additionally, effective phosphorus and effective nitrogen significantly impacted the distribution of *S. alopecuroides*, with the probability of presence being greatest at effective phosphorus levels of 6 mg/kg and effective nitrogen levels of 53 mg/kg. *S. salsula* demonstrated specific soil requirements, with the probability of species presence peaking when the effective phosphorus concentration in the soil was 11 mg/kg (Figure 5, Figures S1–S8).

### 3.4 Niche Breadth and Niche Overlap in Eight Species of Leguminosae

**FIGURE 6:**
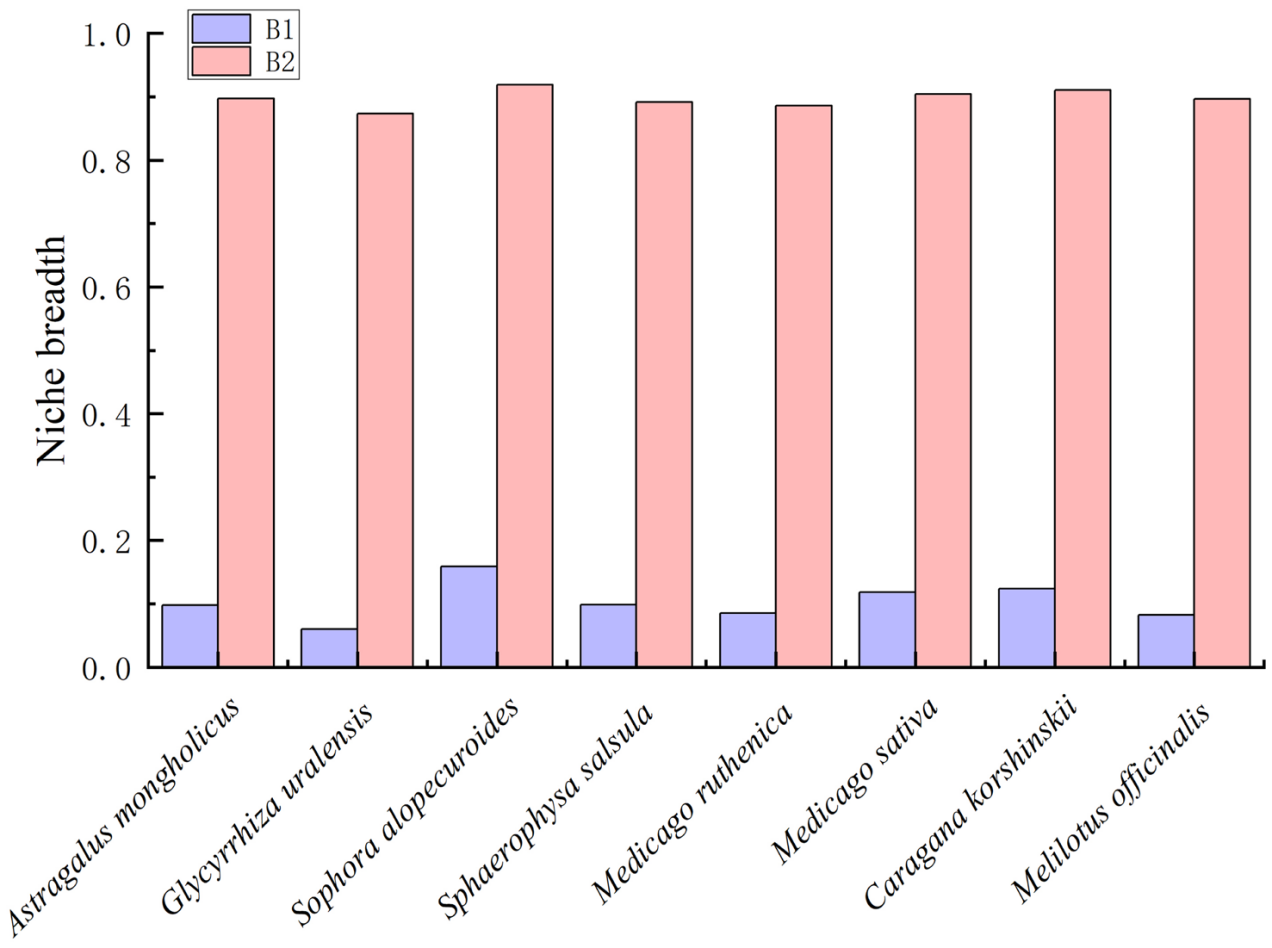
Niche breadth of eight species of Leguminosae (B1 and B2).

By analyzing the niche breadth, niche overlap, and range overlap, we found that the ecotopes of *S. alopecuroides*, *C. korshinskii*, and *M. sativa* were wider, with *S. alopecuroides* exhibiting the highest concentrations of B1 and B2, measured at 0.16 and 0.92, respectively. This was followed by *A. mongholicus*, *M. officinalis*, *S. salsula*, *M. ruthenica*, and *G. uralensis*, which had values of 0.06 and 0.87 for B1 and B2, respectively, as illustrated in Figure 6 and Table S4. The results of ecotope overlap indicated that among different habitats, *M. sativa* exhibited the highest ecotope overlap with itself (*D* = 0.69, *I* = 0.92), followed by *M. ruthenica* and *M. officinalis* (*D* = 0.65, *I* = 0.88). The lowest ecotope overlap was observed between *M. sativa* and *S. salsula* (*D* = 0.40, *I* = 0.67), as shown in Table 3. Regarding range overlap, *G. uralensis* demonstrated a significant degree of overlap with most species, achieving an overlap value of 0.81 with *S. salsula*, followed closely by *M. sativa* and *M. officinalis*, which had a range overlap value of 0.80. Conversely, *S. salsula* exhibited relatively low range overlap with most species, particularly with *M. sativa*, *M. officinalis*, and *S. alopecuroides*, which had range overlap values of 0.25, 0.30, and 0.32, respectively (Figure 6, Table S5).

**TABLE 3:** Niche overlap of potential habitat areas of eight species of Leguminosae (AM, *A. mongholicus*; GU, *G. uralensis*; SA, *S. alopecuroides*; SS, *S. salsula*; MR, *M. ruthenica*; MS, *M. sativa*; CK, *C. korshinskii*; MO, *M. officinalis*).

D (above the diagonal)/I (below the diagonal)	AM	GU	SA	SS	MR	MS	CK	MO
AM	1	0.51	0.47	0.44	0.63	0.56	0.56	0.54
GU	0.77	1	0.49	0.58	0.55	0.44	0.52	0.47
SA	0.75	0.79	1	0.50	0.56	0.60	0.56	0.49
SS	0.69	0.85	0.78	1	0.45	0.39	0.51	0.40
MR	0.83	0.58	0.83	0.72	1	0.69	0.57	0.65
MS	0.80	0.74	0.85	0.67	0.92	1	0.57	0.60
CK	0.82	0.80	0.83	0.79	0.81	0.85	1	0.53
MO	0.79	0.78	0.78	0.71	0.86	0.88	0.80	1

### 3.5 Future Distribution Prediction and Fluctuation Analysis of Suitable Habitats

**FIGURE 7:**
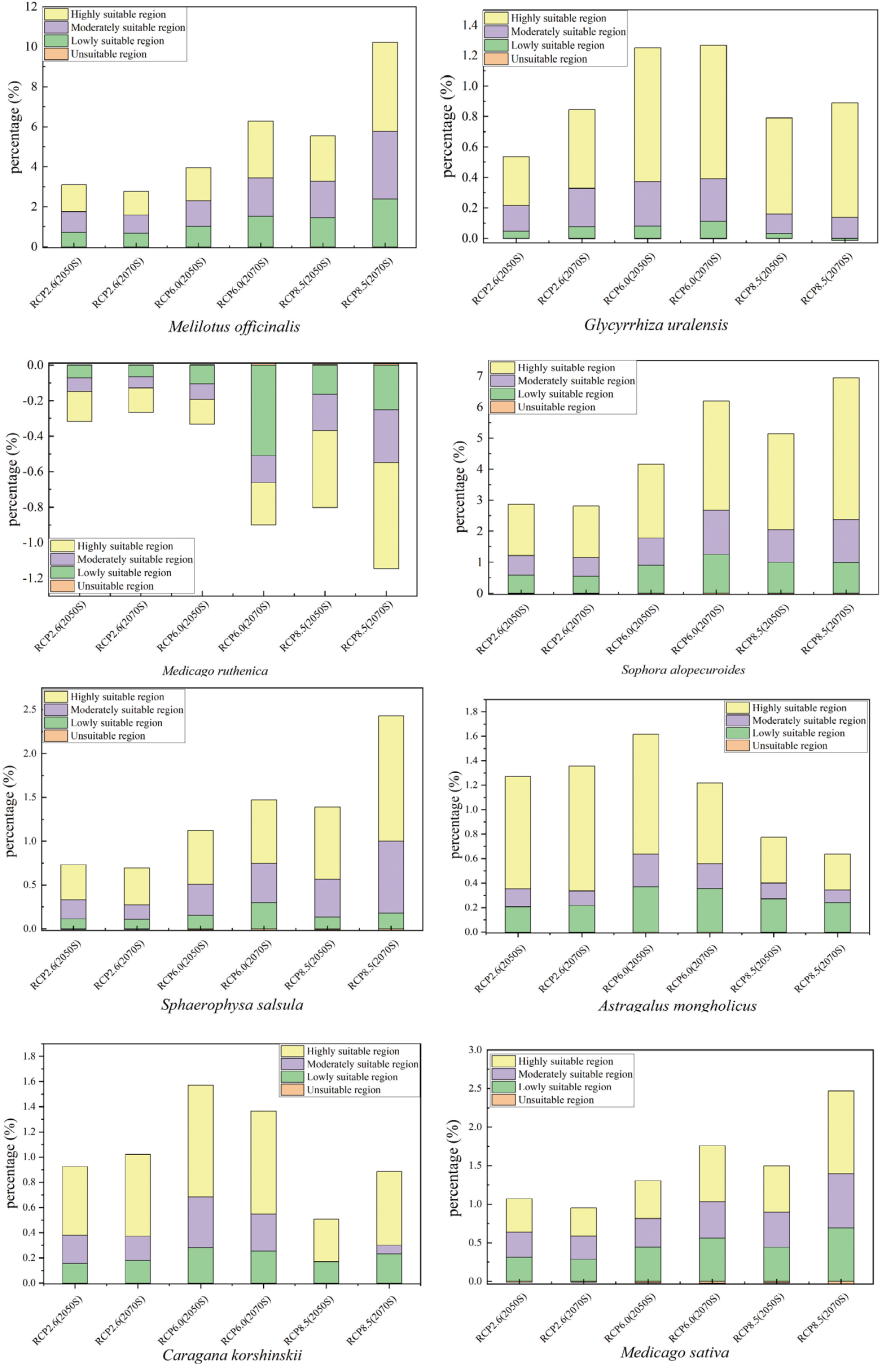
Four different levels of fluctuation in China's future climate scenarios: Green, purple, yellow and orange represent the proportion of low, medium and high suitability areas and unsuitable habitats, respectively. A positive value indicates an increase in area and a negative value indicates a decrease in area.

Future climate model projections indicate a decrease in suitable habitat for *M. ruthenica* across all future climate scenarios of global warming, while suitable habitat for the other seven species of Leguminosae is predicted to increase. Specific data are presented in Table S3. Compared to current projections, the fluctuations in the medium and high suitability zones for *M. ruthenica* are −18.0% and −36.7%, respectively. The reduction in habitat for *M. ruthenica* intensifies as greenhouse gas (GHG) concentrations rise. Notably, habitat reduction in 2050 is more pronounced than in 2070 under the RCP2.6 scenario, whereas habitat reduction in 2070 exceeds that of 2050 under all other CO_2_ scenarios. This suggests that the suitable habitat for *M. ruthenica* on the Qinghai‐Xizang Plateau will diminish under future climate scenarios. Conversely, the most significant increases were observed for *M. officinalis* and *S. alopecuroides*, with fluctuations of 101.6% and 310.7% in the medium and high suitable zones, respectively, for *S. alopecuroides*, and 215.3% and 288.5% in the medium suitable zone for *M. officinalis*. The growth of both *M. officinalis* and *S. alopecuroides* progressively increases with rising GHG concentrations. The expansion of suitable habitats occurs over time, with the exception that in the RCP 2.6 scenario, the growth in 2050 is greater than that in 2070 (Figure 7).

**FIGURE 8:**
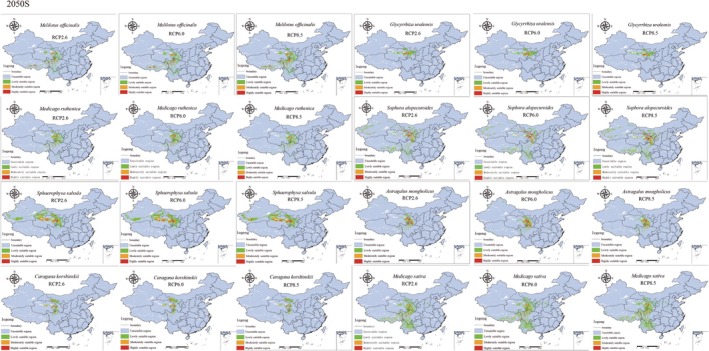
Prediction of the suitable range of the eight species of Leguminosae in the 2050s. RCP representative concentration pathway.

**FIGURE 9:**
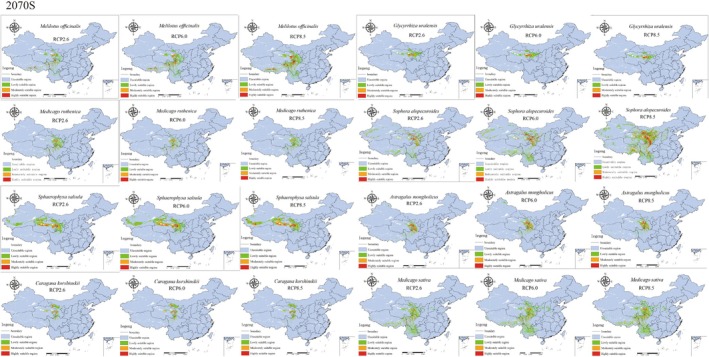
Prediction of the suitable range of the eight species of Leguminosae in the 2070s. RCP representative concentration pathway.

The trends in suitable habitat growth for *S. salsula* and *M. sativa* were similar to those observed for *M. officinalis* and *S. alopecuroides*, albeit with a comparatively smaller magnitude of growth. *G. uralensis*, *A. mongholicus*, and *C. korshinskii* exhibited the most significant increase in suitable habitat under the RCP 6.0 scenario. However, in the RCP 8.5 scenario, the habitat growth for these three Leguminosae species declined. Notably, the growth of *G. uralensis* in 2050 was less than that in 2070, although there was a slight increase in suitable habitat growth for this species over time. In contrast, *A. mongholicus* and *C. korshinskii* demonstrated greater suitable habitat growth in 2050 compared to 2070 across all CO_2_ concentrations, except in the RCP 2.6 scenario, where the growth in 2050 was lower than that in 2070. This indicates that the suitable habitats for *G. uralensis*, *A. mongholicus*, and *C. korshinskii* are likely to trend downward under future climate scenarios (Figure 8, Figure 9).

## 4 Discussion

The Leguminosae species selected for this study have a wide range of applications and are very valuable resources. According to previous studies, not many studies have been conducted on the distribution of the Leguminosae species selected for this study on the Qinghai‐Xizang Plateau. Understanding the suitable habitats of Leguminosae species under the Qinghai‐Xizang Plateau can provide an important basis for their conservation (Shi et al. 2017).

### 4.1 Analysis of Key Environmental Variables

Among the 28 environmental variables, altitude is the most significant factor influencing the distribution model of Leguminosae species, indicating its critical role in their distribution (Zhang, Zhang, et al. 2018; Zhang, Yao, et al. 2018). Downie (2014) has consistently shown that altitude significantly affects alpine species on the Qinghai‐Xizang Plateau. WANG and colleagues have suggested that global warming may lead to a drastic decline or even extinction of species adapted to low temperatures, as alpine plants are specifically suited to survive in such environments (Wang et al. 2023). Additionally, ZHANG and colleagues have proposed that alpine plants may adapt to climate change by adjusting their ecological niches or migrating to higher altitudes and latitudes (Zhang, Zhang, et al. 2018; Zhang, Yao, et al. 2018). According to the findings of the present study, *S. alopecuroides* and *C. korshinskii* are suitable for cultivation at lower altitudes of 2000 m and below, while *A. mongholicus*, *G. uralensis*, and *M. ruthenica* thrive at altitudes ranging from 2300 to 2550 m. Conversely, *S. salsula*, *M. sativa*, and *M. officinalis* are well‐suited for high altitudes above 3000 m. These results indicate that the Leguminosae species examined in this study are adapted to different altitudes. Higher altitudes have a number of elements that are unfavorable to plant growth, such as cooler temperatures and less precipitation. Meanwhile, the relatively high percentages of fat and soluble sugars in seeds at elevated altitudes may provide sufficient energy for seed germination and initial seedling growth, thereby enhancing the seedlings' ability to withstand harsh environmental conditions; therefore, Leguminosae adapted to grow and develop at high altitudes are cold and drought tolerant (Feng et al. 2018).

Differences in altitude mean that they require different temperatures and precipitation for growth. The four species—*A. mongholicus*, *M. sativa*, *M. officinalis*, and *S. alopecuroides*—exhibit the highest probability of occurrence at mean annual temperatures ranging from 6°C to 10°C. This observation suggests that these plants thrive under cooler conditions, while excessive temperatures may hinder their growth. Wang et al. (2015) indicates that *M. sativa* can grow at even lower temperatures, being well adapted to semiarid alpine meadows or alpine grasslands where the mean annual temperature is approximately −1.5°C. *M. ruthenica* is suited to environments with a mean monthly temperature of 15.6°C, while *C. korshinskii* thrives best at mean monthly temperatures between 15°C and 16°C, indicating that both species are adapted to milder climates. Furthermore, MA and colleagues have proposed that low temperatures are among the most significant limiting factors affecting the phenology of alpine plants (Ma and Sun 2018). Additionally, YANG and colleagues have identified rainfall as a crucial factor influencing the functioning and structure of plants and terrestrial ecosystems on the Qinghai‐Xizang Plateau (Yang et al. 2015). The coexistence of *A. mongholicus*, *Medicago sativa* , *M. officinalis*, and *M. ruthenica* is most probable in regions with annual precipitation levels of 600 mm to 750 mm, suggesting that these species inhabit humid areas where moisture availability may limit their growth. In contrast, *C. korshinskii* is a typical arid plant, thriving best in regions with an annual precipitation of 100 mm, a finding that aligns with previous studies (Zhang et al. 2009).

Additionally, *M. sativa* exhibits the highest probability of presence when isothermality reaches 47%. The probability of presence for *S. salsula* and *G. uralensis* is maximized at isothermality levels of 34.5% and 36%, respectively. HONG and his colleagues have proposed that a high isothermality allows alpine plants to capitalize on relatively high daytime temperatures for photosynthesis, while cooler nighttime temperatures reduce respiratory energy consumption. This dynamic favors the accumulation of nutrients in their fusiform, fleshy root systems, thereby facilitating plant growth (Hong et al. 2017).

Based on the findings of this research, the impact of soil factors on Leguminosae was minimal, with the highest likelihood of *S. alopecuroides* occurrence at effective phosphorus levels of 6 mg/kg and effective nitrogen levels of 53 mg/kg. Conversely, the presence of *S. salsula* peaked at an effective phosphorus concentration of 11 mg/kg. *S. salsula* and *S. alopecuroides* exhibit distinct soil nutrient needs. Each Leguminosae species possesses some capacity for nitrogen fixation and can form symbiotic associations with nitrogen‐fixing bacteria found in the soil, leading to the development of rhizomes for nitrogen utilization in the environment. The nutritional requirements of Leguminosae related to soil will be investigated more thoroughly in future research (Yang et al. 2022).

### 4.2 Analysis of Niche Breadth and Niche Overlap

A notable correlation exists between the width of ecotopes and species range (Slatyer et al. 2013). Species that occupy wider ecotopes possess a larger area classified as suitable habitat, leading to greater potential geographic ranges (Sheth et al. 2013). Earlier research indicates that assessing the ecotope width for various species is crucial for prioritizing them in conservation efforts (Savolainen et al. 2013). In our study, we observed that *S. alopecuroides*, *C. korshinskii*, and *M. sativa* exhibited wider ecotopes, while *G. uralensis* was found to have narrower ones, aligning with previous findings. In stable ecosystems, ecological niche overlap among closely related species can lead to competition for habitat, and species that share limited resources along similar ecological niches are unlikely to persist together over extended durations (Anacker and Strauss 2014). Therefore, examining the extent of ecological niche and range overlap among species is essential for understanding their interrelationships. Our findings revealed the highest degree of ecological niche overlap occurred between *M. sativa* and *M. ruthenica*, as well as *M. officinalis*, whereas the overlap was minimal when comparing *M. sativa* and *S. salsula*. This suggests a significant level of competition between *M. sativa* and *M. ruthenica*, as well as *M. officinalis*, while *M. sativa* and *S. salsula* exhibit the least competition for habitat. Regarding range overlap, *G. uralensis* demonstrated considerable overlap with most species, while *S. salsula* showed relatively limited range overlap with the majority. This implies that *G. uralensis* shares a similar habitat space with other species in this investigation, while *S. salsula* occupies a more distinct habitat.

### 4.3 Changes in Distribution of Leguminosae in the Future

The Qinghai‐Xizang Plateau is home to numerous plant species that have adapted to low‐temperature conditions, yet their habitats are projected to diminish to varying extents due to the intensifying impacts of global warming (Wang et al. 2019). Research conducted by JIANG and colleagues assessed the potential habitat ranges for three mussel species on the Qinghai‐Xizang Plateau, revealing that these habitats are anticipated to reduce in future climate change scenarios (Jiang et al. 2022). Conversely, certain species may benefit from their superior adaptive capacity to environmental stressors, leading to an increase in their habitat area under projected global warming conditions (Wu 2011). In another study by YANG and collaborators, the potential habitats for 12 threatened medicinal plants on the Tibetan Plateau were examined. Their findings indicated that, under future scenarios, one‐fourth of these plants would suffer a decline in suitable habitats, while one‐third could see an increase (Yang et al. 2024). This current study indicates a decreasing trend in the future suitable habitat for *M. ruthenica*, whereas the other seven Leguminosae species are experiencing an increase in their habitable areas to different extents. These findings suggest that Leguminosae species may possess a greater adaptability to temperature fluctuations. The most significant habitat expansion was noted in *M. officinalis* and *S. alopecuroides*, while the trends for *S. salsula* and *M. sativa* mirrored those of *M. officinalis* and *S. alopecuroides*, albeit with a smaller relative increase. Under the RCP8.5 scenario, there was a decline in the habitat areas for *G. uralensis*, *A. mongholicus*, and *C. korshinskii*. This implies that, should CO_2_ concentrations remain sufficiently high, the suitable habitats for these three Leguminosae species are likely to trend downward. Temperature serves as a critical constraint on the growth and distribution of alpine plants, and the anticipated rise in global temperatures will significantly influence their distribution in the future.

## 5 Conclusion

In this research, we focused on the Qinghai‐Xizang Plateau as our study site and employed ecological niche theory to forecast both the current and future suitable areas, as well as the ecological niche overlap among eight Leguminosae species within the Qinghai‐Xizang Plateau. Our findings revealed that the distribution ranges of *M. officinalis*, *M. ruthenica*, *S. alopecuroides*, *M. sativa*, and *S. salsula* were extensive, while *C. korshinskii* exhibited the smallest suitable area. The distribution patterns of these eight Leguminosae plants were predominantly influenced by altitude, aligning with the conclusions drawn in most existing studies concerning flora on the Qinghai‐Xizang Plateau. Notably, *S. alopecuroides* had the widest ecotope breadth; this provides a link to explaining the size of the species' ranges. Additionally, *M. sativa* and *M. ruthenica* displayed the highest degree of ecotope overlap, suggesting a strong connection between them. Except for *M. ruthenica*, the areas of suitable habitats for the other seven Leguminosae species are projected to expand in the future, indicating that the Leguminosae studied are well‐equipped to adapt to environmental changes on the Qinghai‐Xizang Plateau.

## Author Contributions

**Sen‐Xin Chai:** formal analysis (lead), investigation (lead), visualization (lead), writing – original draft (lead). **Hui‐Yuan Ma:** investigation (supporting), software (supporting). **Chen‐Di Wang:** investigation (supporting). **Yan‐Gang Ying:** investigation (supporting). **Dong Han:** investigation (supporting). **Yue Zhong:** investigation (supporting). **Bo Wang:** conceptualization (lead), methodology (lead), software (supporting). **Yuan‐Ming Xiao:** conceptualization (supporting), methodology (supporting), software (supporting). **Ying Yang:** methodology (supporting). **Guo‐Ying Zhou:** conceptualization (lead), funding acquisition (lead), methodology (lead), writing – review and editing (supporting).

## Conflicts of Interest

The authors declare no conflicts of interest.

## Supporting information


**Data S1:** ece371895‐sup‐0001‐DataS1.docx.

**Data S2:** ece371895‐sup‐0002‐DataS2.xlsx.

## Data Availability

We used open‐access data from the CNKI database (http://www.cnki.net/ accessed on 7 October 2022), China Digital Herbarium (https://www.cvh.ac.cn/ accessed on 7 October 2022), NSII China National Herbarium Resource Leveling (http://www.nsii.org.cn/ 2017/home.php accessed on 7 October 2022), Global Biodiversity Information Facility (https://www.gbif.org/zh/), China Plant Image Library (http://ppbc.iplant.cn/ accessed on 7 October 2022); readers can find the occurrence data in the Supporting Information.
