# Supplementary material for: Combining Niche Breadth to Predict the Current and Future Distribution of Leguminosae Under Climate Change on the Qinghai‐Xizang Plateau
Source: Ecol Evol. 2025 Aug 21;15(8):e71895. doi: 10.1002/ece3.71895 (PMC12370446; doi:10.1002/ece3.71895)
Supplement: Supplementary file 1 — Data S1: ece371895‐sup‐0001‐DataS1.docx. [file ECE3-15-e71895-s002.docx]

**Supplementary Material**

Combining niche breadth to Predict the Current and Future Distribution of *Leguminosae* under Climate Change on the Qinghai-Xizang Plateau

Sen-Xin Chai[^1^](#OLE_LINK12)^,^[^2^](#OLE_LINK13), Hui-yuan Ma[^1^](#OLE_LINK12)^,^[^2^](#OLE_LINK13), Chen-Di Wang^5^, Yan-Gang Ying^5^, Dong Han^5^, Yue Zhong^5^, Bo Wang^1,3^, Yuan-Ming Xiao^1,3^, Ying Yang^1,4^ and Guo-Ying Zhou^1^ [^*^](#通讯)

^1^ CAS Key Laboratory of Tibetan Medicine Research, Northwest Institute of Plateau Biology, Xining 810008, China

^2^ College of Life Sciences, Qinghai Normal University, Xining 810016, China

^3^ University of Chinese Academy of Sciences, Beijing 100049, China

^4^ Qinghai University, Xining 810016, China

^5^Qinghai Traffic Construction Management Co., Ltd., Xining,810000 China

*Corresponding author: Dr. Guoying Zhou, Tel: +86-971-6159630, Fax: +86-971-

6143282, E-mail: zhougy@nwipb.cas.cn

ORCID: https://orcid.org/0000-0003-2485-6172

Address: 23# Xinning Road, Xining, Qinghai, P. R. China 810008

**Supplementary figures**

**
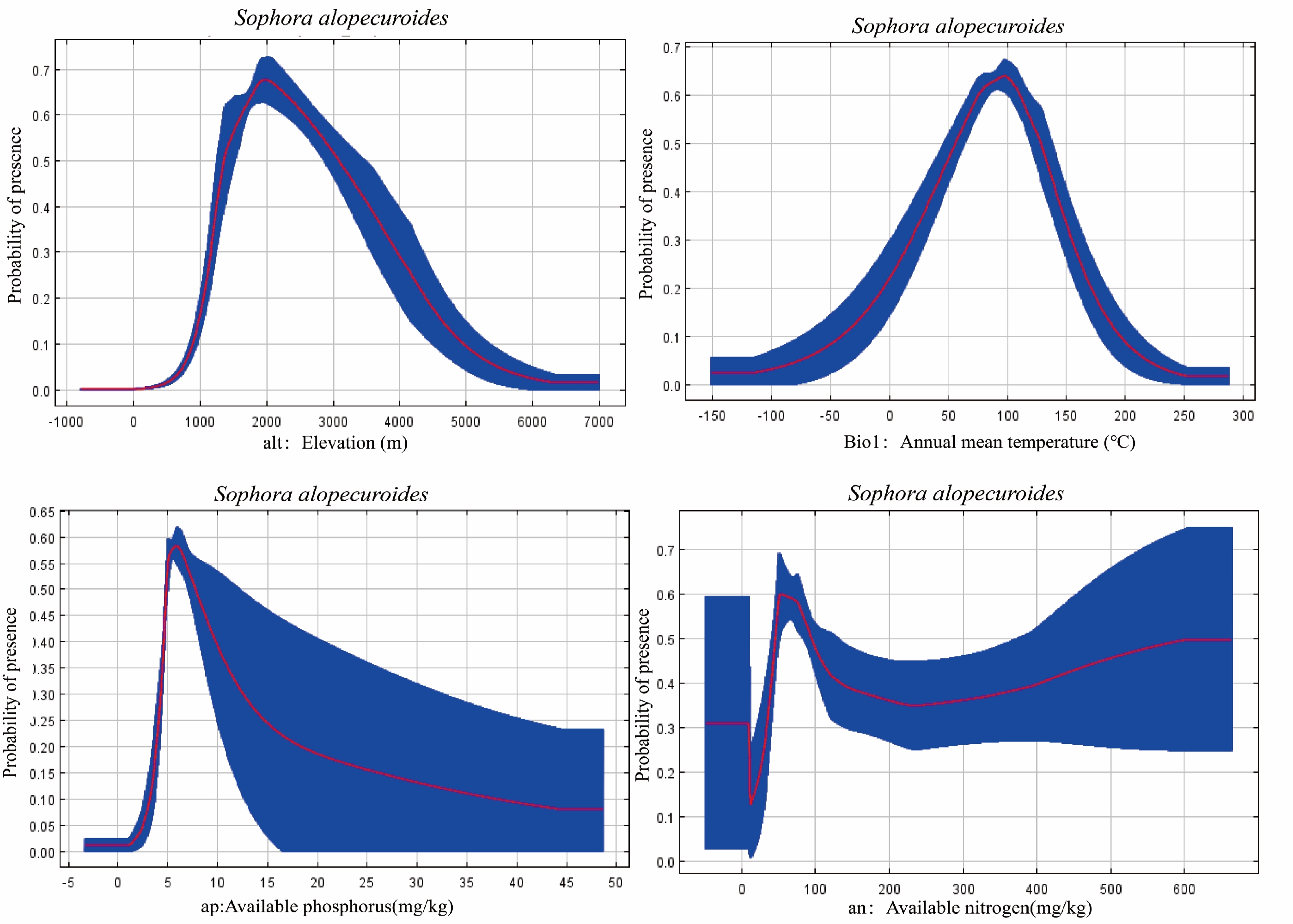
**

**Figure. S1** Response curve of the key environmental factors of *Sophora alopecuroides*


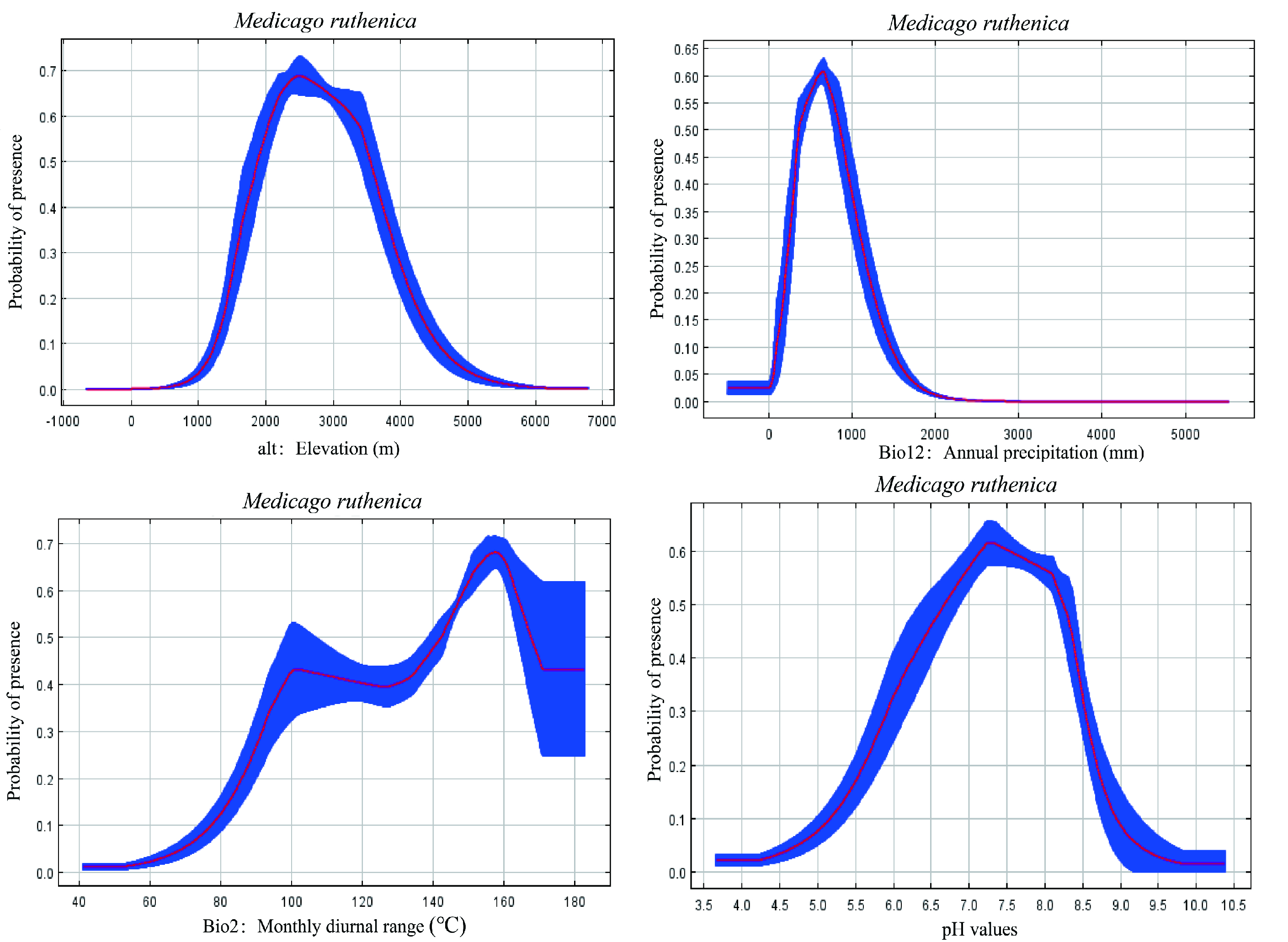


**Figure. S2** Response curve of the key environmental factors of *Medicago ruthenica*


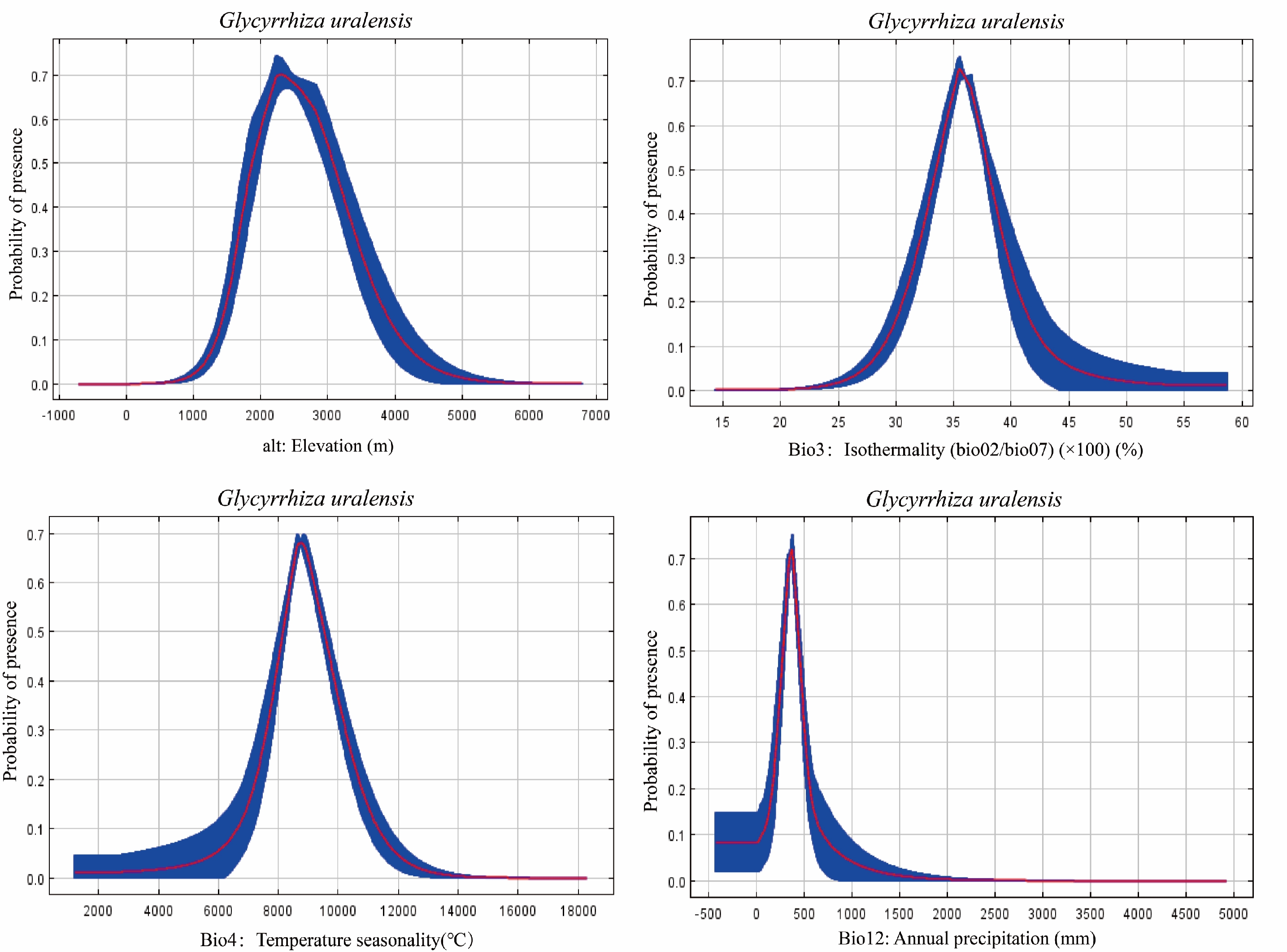


**Figure. S3** Response curve of the key environmental factors of *Glycyrrhiza uralensis*

*
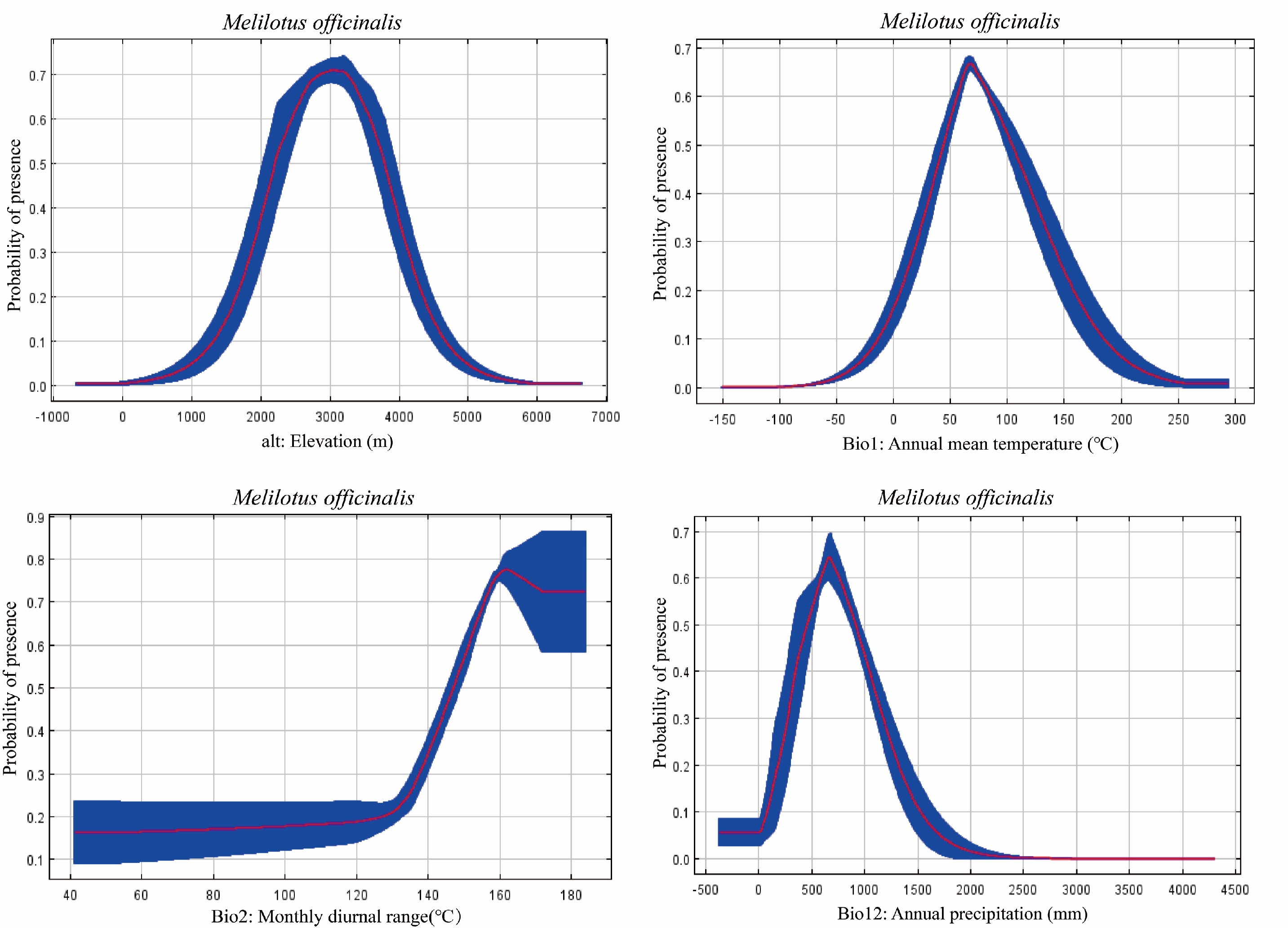
*

**Figure. S4** Response curve of the key environmental factors of *Melilotus officinalis*

*
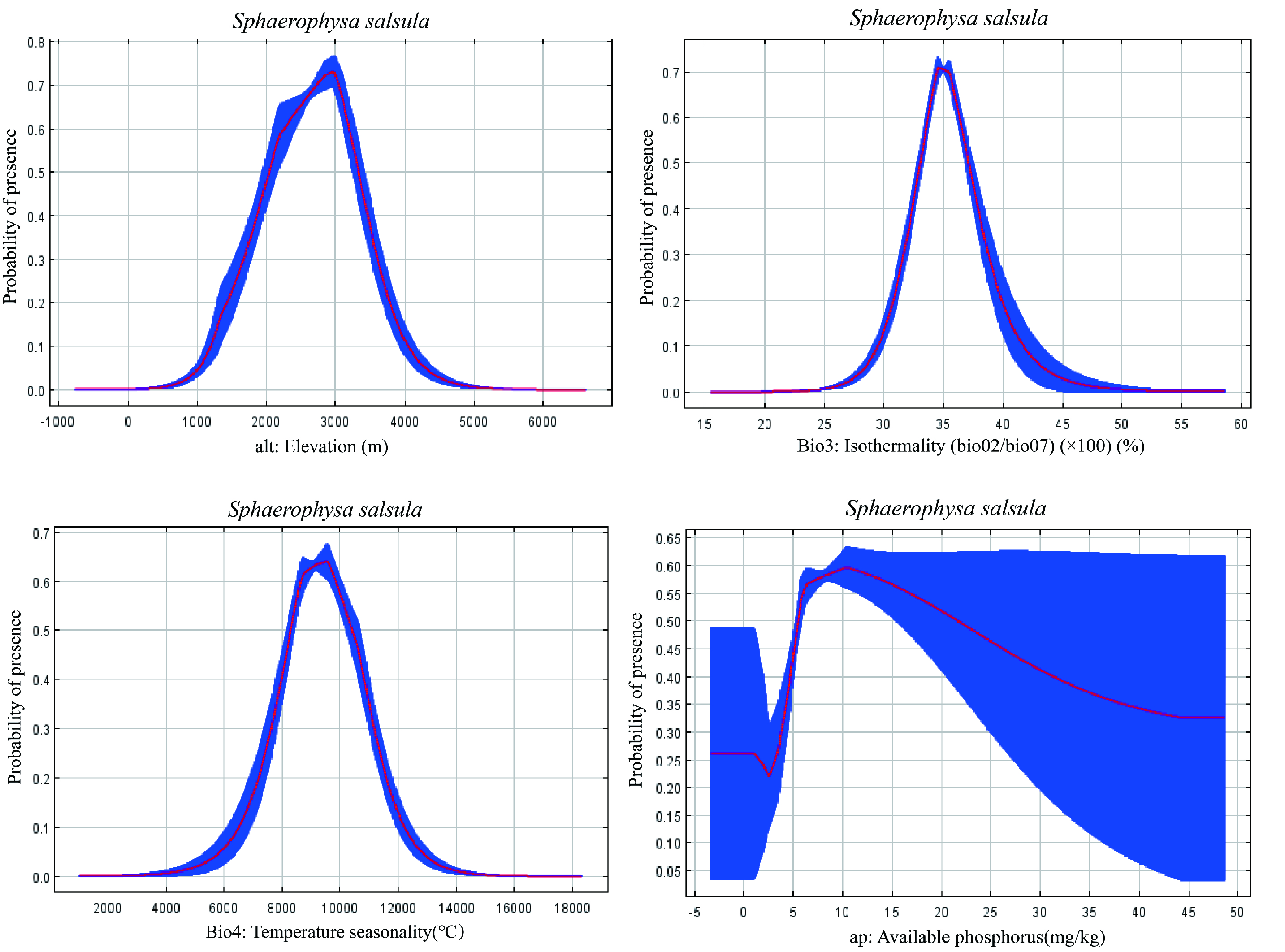
*

**Figure. S5** Response curve of the key environmental factors of *Sphaerophysa salsula*


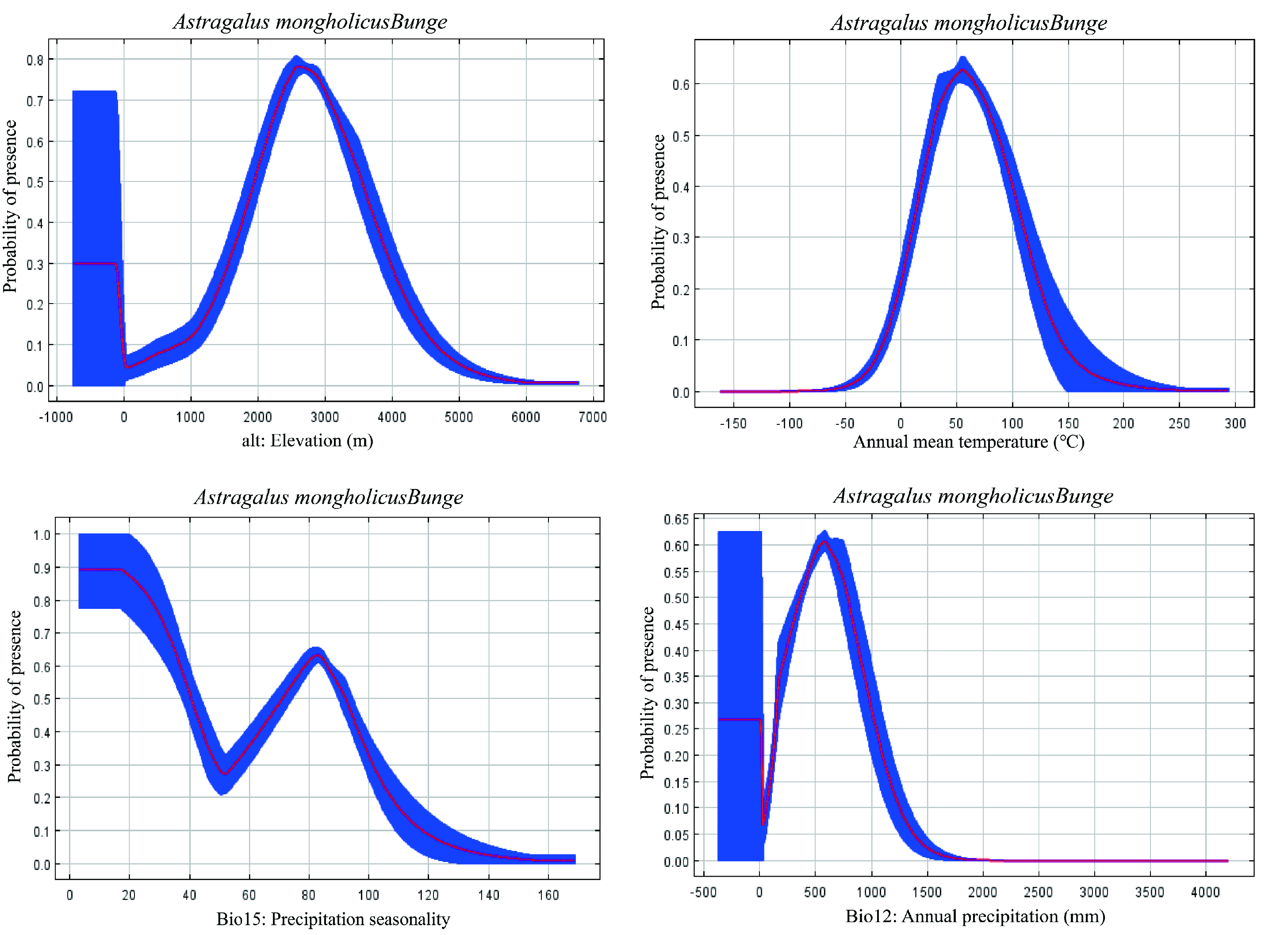


**Figure. S6** Response curve of the key environmental factors of *Astragalus mongholicusBunge*


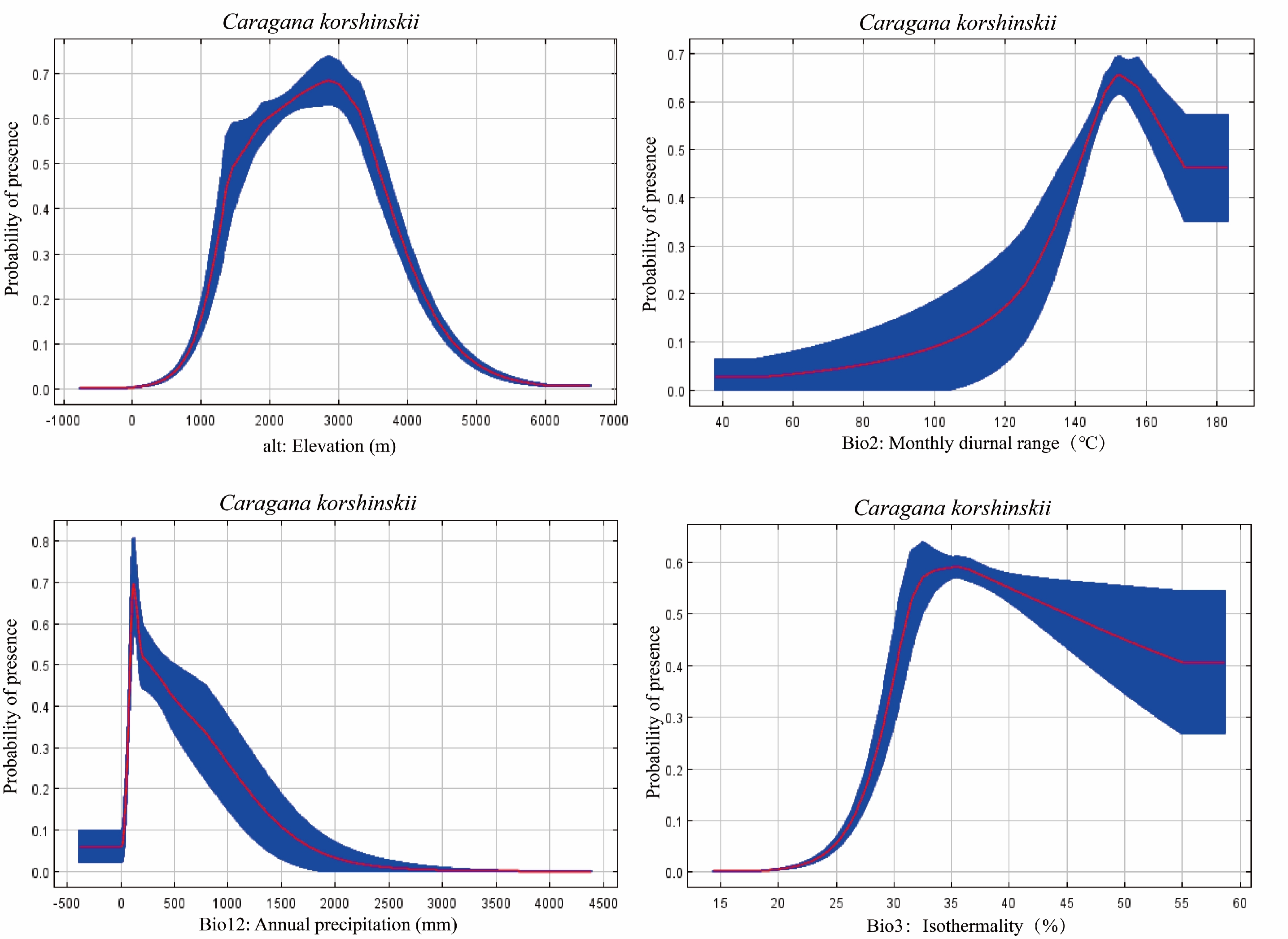


**Figure. S7** Response curve of the key environmental factors of *Caragana korshinskii*


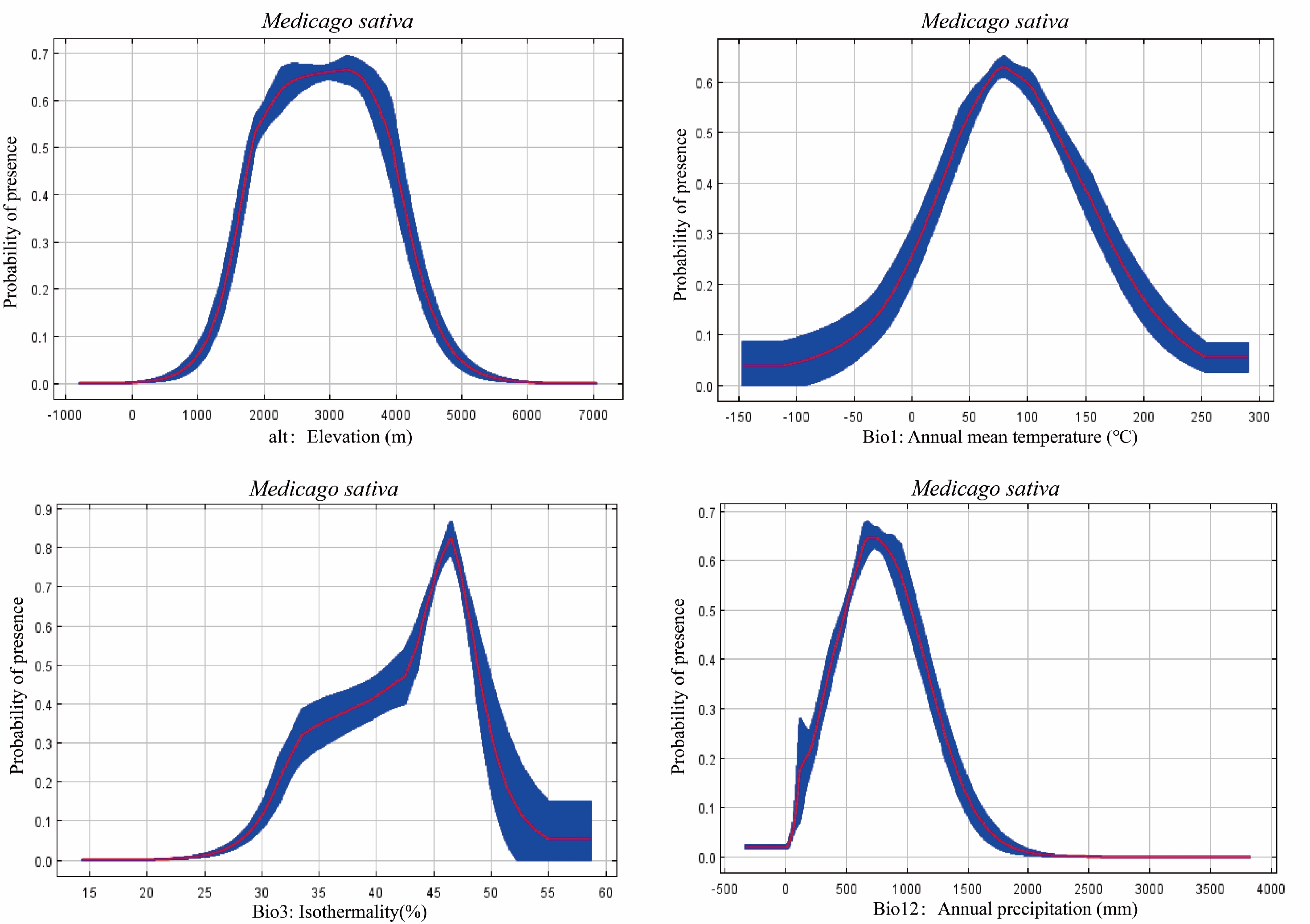


**Figure. S8** Response curve of the key environmental factors of *Medicago sativa*

**Table S1.** List of environmental variables used in this research

| Table S1. 28 environmental variables (19 climate variables, 8 soil variables, 1 topographic variable) | | | |
| --- | --- | --- | --- |
| Type | Variable | Description (Unit) | |
| Bioclimatic variable | Bio1 | Annual mean temperature (◦C) | |
|  | Bio2 | Monthly diurnal range (mean of monthly (max temp − min temp)) (◦C) | |
|  | Bio3 | Isothermality (bio02/bio07) (×100) (◦C) | |
|  | Bio4 | Temperature seasonality (standard deviation × 100) (◦C) | |
|  | Bio5 | Max temperature of the warmest month (◦C) | |
|  | Bio6 | Min temperature of the coldest month (◦C) | |
|  | Bio7 | Temperature annual range (Bio5–Bio6) (◦C) | |
|  | Bio8 | Mean temperature of wettest quarter (◦C) | |
|  | Bio9 | Mean temperature of the driest quarter (◦C) | |
|  | Bio10 | Mean temperature of the warmest quarter (◦C) | |
|  | Bio11 | Mean temperature of the coldest quarter (◦C) | |
|  | Bio12 | Annual precipitation (mm) | |
|  | Bio13 | Precipitation of the wettest month (mm) | |
|  | Bio14 | Precipitation of the driest month (mm) | |
|  | Bio15 | Precipitation seasonality (coefficient of variation) | |
|  | Bio16 | Precipitation of the wettest quarter (mm) | |
|  | Bio17 | Precipitation of the driest quarter (mm) | |
|  | Bio18 | Precipitation of the warmest quarter (mm) | |
|  | Bio19 | Precipitation of the coldest quarter (mm) | |
|  | alt | Elevation (m) | |
| Soil variable | ak | Available potassium(mg/kg) | |
|  | an | Available nitrogen(mg/kg) | |
|  | ap | Available phosphorus(mg/kg) | |
|  | pH | pH values | |
|  | som | organic matter（g/kg） | |
|  | tk | Total potassium(mg/L) | |
|  | tp | Total phosphorus(mg/kg) |  |
|  | tn | Total nitrogen (mg/kg) |  |

**Table S2** Indicators of suitability of eight species of *Leguminosae*

| Species | Unsuitable region | Lowly suitable region | Moderately suitable region | Highly suitable region |
| --- | --- | --- | --- | --- |
| *Sophora alopecuroides* | 0-0.3147 | 0.3147-0.5431 | 0.5431-0.7716 | 0.7716-1 |
| *Medicago ruthenica* | 0-0.2446 | 0.2446-0.4964 | 0.4964-0.7482 | 0.7482-1 |
| *Glycyrrhiza uralensis* | 0-0.1879 | 0.1879-0.4586 | 0.4586-0.7293 | 0.7293-1 |
| *Melilotus officinalis* | 0-0.2085 | 0.2085-0.4723 | 0.4723-0.7362 | 0.7362-1 |
| *Sphaerophysa salsula* | 0-0.1694 | 0.1694-0.4463 | 0.4463-0.7231 | 0.7231-1 |
| *Astragalus mongholicusBung* | 0-0.3314 | 0.3314-0.5543 | 0.5543-0.7771 | 0.7771-1 |
| *Caragana korshinskii* | 0-0.4193 | 0.4193-0.6129 | 0.6129-0.8063 | 0.8063-1 |
| *Medicago sativa* | 0-0.1924 | 0.1924-0.4616 | 0.4616-0.7308 | 0.7308-1 |

**Table S3** Forecast area (×10^4^ km^2^) of suitable habitat

|  |  | Decades | Total suitable region | Lowly suitable region | Moderately suitable region | Highly suitable region |
| --- | --- | --- | --- | --- | --- | --- |
| *Sophora alopecuroides* |  | 1960-1990 | 49.04 | 17.83 | 6.42 | 1.01 |
|  | RCP2.6 | 2050 | 41.40 | 28.26 | 10.48 | 2.67 |
|  |  | 2070 | 40.63 | 27.66 | 10.30 | 2.67 |
|  | RCP6.0 | 2050 | 49.39 | 33.98 | 12.02 | 3.40 |
|  |  | 2070 | 60.20 | 40.07 | 15.59 | 4.54 |
|  | RCP8.5 | 2050 | 52.88 | 35.58 | 13.18 | 4.12 |
|  |  | 2070 | 56.39 | 35.47 | 15.32 | 5.60 |
| *Medicago ruthenica* |  | 1960-1990 | 24.74 | 17.85 | 5.78 | 1.10 |
|  | RCP2.6 | 2050 | 22.81 | 16.55 | 5.34 | 0.92 |
|  |  | 2070 | 23.05 | 16.68 | 5.42 | 0.95 |
|  | RCP6.0 | 2050 | 22.20 | 15.96 | 5.28 | 0.95 |
|  |  | 2070 | 14.50 | 8.76 | 4.89 | 0.84 |
|  | RCP8.5 | 2050 | 20.15 | 14.93 | 4.59 | 0.63 |
|  |  | 2070 | 17.86 | 13.35 | 4.07 | 0.44 |
| *Glycyrrhiza uralensis* |  | 1960-1990 | 15.48 | 12.66 | 2.32 | 0.50 |
|  | RCP2.6 | 2050 | 16.64 | 13.27 | 2.72 | 0.66 |
|  |  | 2070 | 17.27 | 13.60 | 2.91 | 0.75 |
|  | RCP6.0 | 2050 | 17.60 | 13.66 | 3.01 | 0.93 |
|  |  | 2070 | 17.97 | 14.06 | 2.97 | 0.93 |
|  | RCP8.5 | 2050 | 16.49 | 13.06 | 2.62 | 0.81 |
|  |  | 2070 | 16.00 | 12.49 | 2.64 | 0.87 |
| *Melilotus officinalis* |  | 1960-1990 | 14.87 | 11.24 | 2.67 | 0.96 |
|  | RCP2.6 | 2050 | 27.01 | 19.29 | 5.48 | 2.24 |
|  |  | 2070 | 26.01 | 18.78 | 5.14 | 2.09 |
|  | RCP6.0 | 2050 | 31.34 | 22.70 | 6.09 | 2.55 |
|  |  | 2070 | 39.88 | 28.41 | 7.78 | 3.68 |
|  | RCP8.5 | 2050 | 38.31 | 27.63 | 7.57 | 3.12 |
|  |  | 2070 | 55.03 | 38.10 | 11.73 | 5.21 |
| *Sphaerophysa salsula* |  | 1960-1990 | 44.29 | 33.37 | 9.03 | 1.89 |
|  | RCP2.6 | 2050 | 50.82 | 37.19 | 10.98 | 2.65 |
|  |  | 2070 | 50.15 | 36.94 | 10.50 | 2.70 |
|  | RCP6.0 | 2050 | 53.87 | 38.61 | 12.20 | 3.06 |
|  |  | 2070 | 59.67 | 43.34 | 13.06 | 3.27 |
|  | RCP8.5 | 2050 | 54.24 | 37.89 | 12.89 | 3.46 |
|  |  | 2070 | 60.33 | 39.27 | 16.45 | 4.60 |
| *Astragalus mongholicusBunge* |  | 1960-1990 | 15.24 | 8.77 | 5.28 | 1.19 |
|  | RCP2.6 | 2050 | 18.92 | 10.58 | 6.05 | 2.29 |

**continued form Table S3** Forecast area (×10^4^ km^2^) of suitable habitat

|  |  | Decades | Total suitable region | Lowly suitable region | Moderately suitable region | Highly suitable region |
| --- | --- | --- | --- | --- | --- | --- |
|  |  | 2070 | 19.00 | 10.68 | 5.91 | 2.41 |
|  | RCP6.0 | 2050 | 21.06 | 12.01 | 6.69 | 2.36 |
|  |  | 2070 | 20.22 | 11.89 | 6.34 | 1.98 |
|  | RCP8.5 | 2050 | 18.75 | 11.15 | 5.95 | 1.64 |
|  |  | 2070 | 18.25 | 10.89 | 5.82 | 1.54 |
| *Caragana korshinskii* |  | 1960-1990 | 13.06 | 8.39 | 4.18 | 0.49 |
|  | RCP2.6 | 2050 | 15.57 | 9.69 | 5.12 | 0.76 |
|  |  | 2070 | 15.70 | 9.92 | 4.97 | 0.81 |
|  | RCP6.0 | 2050 | 17.53 | 10.73 | 5.87 | 0.93 |
|  |  | 2070 | 16.83 | 10.54 | 5.40 | 0.89 |
|  | RCP8.5 | 2050 | 14.66 | 9.81 | 4.19 | 0.66 |
|  |  | 2070 | 15.58 | 10.33 | 4.47 | 0.78 |
| *Medicago sativa* |  | 1960-1990 | 39.36 | 30.37 | 7.75 | 1.23 |
|  | RCP2.6 | 2050 | 51.82 | 39.76 | 10.29 | 1.77 |
|  |  | 2070 | 50.84 | 39.06 | 10.09 | 1.68 |
|  | RCP6.0 | 2050 | 56.37 | 43.90 | 10.62 | 1.84 |
|  |  | 2070 | 60.95 | 47.43 | 11.38 | 2.13 |
|  | RCP8.5 | 2050 | 57.07 | 43.79 | 11.31 | 1.97 |
|  |  | 2070 | 67.17 | 51.43 | 13.18 | 2.56 |

**Table S4** Niche breadth (B1 and B2) of eight *Leguminosae* species.

| species | B1 (inverse concentration) | B2 (uncertainty) |
| --- | --- | --- |
| *Astragalus mongholicusBunge* | 0.09798 | 0.89685 |
| *Glycyrrhiza uralensis* | 0.0598 | 0.87346 |
| *Sophora alopecuroides* | 0.15913 | 0.91874 |
| *Sphaerophysa salsula* | 0.09875 | 0.8914 |
| *Medicago ruthenica* | 0.08513 | 0.88595 |
| *Medicago sativa* | 0.11819 | 0.90401 |
| *Caragana korshinskii* | 0.12383 | 0.9104 |
| *Melilotus officinalis* | 0.0828 | 0.8967 |

**Table S5** Niche overlap, range overlap between the two species.

| Species 1 | Species 2 | D_overlap | I_overlap | Rang overlap |
| --- | --- | --- | --- | --- |
| *Astragalus_mongholicusBunge* | *Melilotus_officinalis* | 0.544673 | 0.79380198 | 0.666051299 |
| *Astragalus_mongholicusBunge* | *Glycyrrhiza_uralensis* | 0.505173391 | 0.773744911 | 0.603801141 |
| *Astragalus_mongholicusBunge* | *Medicago_ruthenica* | 0.626697231 | 0.830282745 | 0.694796239 |
| *Astragalus_mongholicusBunge* | *Sophora_alopecuroides* | 0.46899542 | 0.746391404 | 0.446611551 |
| *Astragalus_mongholicusBunge* | *Sphaerophysa_salsula* | 0.440216029 | 0.687833502 | 0.43486374 |
| *Astragalus_mongholicusBunge* | *Caragana_korshinskii* | 0.562850154 | 0.823676242 | 0.575476444 |
| *Astragalus_mongholicusBunge* | *Medicago_sativa* | 0.560994303 | 0.795150419 | 0.600193092 |
| *Caragana_korshinskii* | *Glycyrrhiza_uralensis* | 0.516504882 | 0.802686118 | 0.636367153 |
| *Caragana_korshinskii* | *Melilotus_officinalis* | 0.528197041 | 0.802096144 | 0.60280183 |
| *Caragana_korshinskii* | *Medicago_ruthenica* | 0.567566814 | 0.811305885 | 0.554925497 |
| *Caragana_korshinskii* | *Sophora_alopecuroides* | 0.557243043 | 0.830574354 | 0.30046984 |
| *Caragana_korshinskii* | *Sphaerophysa_salsula* | 0.512417305 | 0.786998623 | 0.437459497 |
| *Caragana_korshinskii* | *Medicago_sativa* | 0.573810232 | 0.851135837 | 0.422759867 |
| *Glycyrrhiza_uralensis* | *Melilotus_officinalis* | 0.466643624 | 0.779527409 | 0.251939259 |
| *Glycyrrhiza_uralensis* | *Medicago_ruthenica* | 0.549202996 | 0.831714179 | 0.639036657 |
| *Glycyrrhiza_uralensis* | *Sophora_alopecuroides* | 0.494555476 | 0.785553089 | 0.599516394 |
| *Glycyrrhiza_uralensis* | *Sphaerophysa_salsula* | 0.576692217 | 0.850126603 | 0.81124867 |
| *Glycyrrhiza_uralensis* | *Medicago_sativa* | 0.440745942 | 0.742916135 | 0.457413676 |
| *Medicago_ruthenica* | *Melilotus_officinalis* | 0.598931953 | 0.858098654 | 0.645245875 |
| *Medicago_ruthenica* | *Sophora_alopecuroides* | 0.5649401 | 0.82534754 | 0.554455148 |
| *Medicago_ruthenica* | *Sphaerophysa_salsula* | 0.44909403 | 0.722416584 | 0.371827965 |
| *Medicago_ruthenica* | *Medicago_sativa* | 0.687629861 | 0.916079576 | 0.629259482 |
| *Medicago_sativa* | *Melilotus_officinalis* | 0.645038296 | 0.881873597 | 0.79702663 |
| *Medicago_sativa* | *Sophora_alopecuroides* | 0.601742276 | 0.852692409 | 0.517119006 |
| *Medicago_sativa* | *Sphaerophysa_salsula* | 0.389066236 | 0.674048088 | 0.250204606 |
| *Melilotus_officinalis* | *Sophora_alopecuroides* | 0.494780228 | 0.781792493 | 0.440834831 |
| *Melilotus_officinalis* | *Sphaerophysa_salsula* | 0.399995675 | 0.706279809 | 0.304116974 |
| *Sophora_alopecuroides* | *Sphaerophysa_salsula* | 0.50106519 | 0.783091809 | 0.321618811 |
